# Analysis on vertical–pitch coupled dynamics characteristics of shearer with corrected load

**DOI:** 10.1038/s41598-021-98221-3

**Published:** 2021-09-20

**Authors:** Xinwei Yang, Hongyue Chen, Pengfei Li, Xin Wang, Yajing Wei

**Affiliations:** 1 Faculty of Electrical and Control Engineering, Liaoning Technical University, Huludao, 125105 Liaoning China; 2 School of Mechanical Engineering, Liaoning Technical University, Fuxin, 123000 Liaoning China; 3 Research Institute of Technology and Equipment for the Exploitation and Utilization of Mineral Resources, Liaoning Technical University, Fuxin, 123000 Liaoning China; 4 Fuxin City Industrial Technology Research Institute, Fuxin, 123000 Liaoning China

**Keywords:** Engineering, Physics

## Abstract

With regard to the severe vertical and pitching vibration of shearer in actual working conditions, the vertical–pitch coupled dynamics model of shearer with 13 degrees of freedom was established using multi-body dynamics theory. Based on the elasticity theory, Hertz contact and collision dynamics, the connection stiffness model of shear body and the traction section, the rocker arm with clearance and the traction section, the support stiffness models for the guide shoe and the smooth shoe, and the stiffness characteristics model of the rocker arm were constructed respectively. Taking into account the convergence of solution process and the reliability of results, a modified drum load was proposed as the external excitation of the system and the dynamic characteristics of key parts of shearer under different working conditions were analyzed. The numerical models were validated against the vibration responses of the whole machine and critical components of shearer obtained from mechanical test under different operating conditions. The research results provide theoretical basis for the structure optimization and process parameter optimization of shearer.

## Introduction

At present stage, coal mining methods are developing from mechanization to intelligent and unmanned. As the core equipment of coal mining, the dynamic characteristics and reliability of shearer directly affect the intelligent and unmanned development process for fully mechanized coal face. In recent years, experts and researchers from countries have done a lot of research on reliability and vibration characteristics of the whole shearer and its key components. Zhang^1,2^ obtained the vibration characteristic curve of the walking part gear transmission system through the dynamic model of the whole coal mining machine, complemented corresponding experimental verification, analyzed the influencing mechanism of varied design parameters on the vibration characteristic curve and obtained the largest deformation and stress position of the walking box. Zhang^3^ used numerical analysis method to analyze the dynamic response of their designed three-drum shearer under different working conditions. Jiang^4,5^ established the electro-mechanical coupling torsional dynamics model for the transmission system of cutting part of the shearer, analyzed the influence of internal control parameters and drum load on the torsional vibration characteristics of the system and obtained the control method of the system in the case of large vibration amplitude and unstable oscillation. Chen^6,7^ established a non-linear dynamics model for the traveling part transmission system of the shearer, obtained the load characteristics of the gears of transmission system and analyzed the dynamic reliability of the gears under different working parameters using fatigue damage theory; the dynamics model of the transmission system of cutting unit was established and the dynamic characteristics of the system under different working temperatures were analyzed. Our previous study^8^ comprehensively considered the connection characteristics of key parts of the shearer, established a traction-swing coupling dynamics model of the shearer, analyzed the dynamic characteristics of those key parts under different working conditions and verified the model and corresponding results by experiment. Hongyue^9^ introduced the experimental load of the drum to analyze the dynamic characteristics and fatigue life of the shearer support mechanism using co-simulation technique. Wang^10^ analyzed the dynamic characteristics of the electro-mechanical coupling system of the shearer cutting unit under random loads. Jia^11,12^ established a coupled dynamics model of the transmission system of cutting part and the rocker arm shell based on an improved gear mechanics model, analyzed the dynamic response of the system under different loads, and obtained influence law of topology optimization of the shell on the characteristics of the gears. Liu^13,14^ proposed a torsional dynamics modeling method for planetary gears, established the electro-mechanical coupling dynamics model for cutting part transmission system and obtained the dynamics response influence law of traction speed and drum load on that system via analysis method. Ge^15^ established the electro-mechanical coupling dynamics model for the cutting unit transmission system, analyzed the dynamic characteristics of the system under impact load and proposed an active control strategy of motor torque compensation to suppress the dynamic loads caused by external loads with catastrophe characteristics. Yang^16^ established the vertical dynamics model for the cutting part of the shearer and analyzed the influence of the support characteristics of the height-adjusting cylinder on the vibration characteristics of the system. Yang et al.^17^ used AMESim software to establish an electro-mechanical coupling dynamics model for the cutting part transmission system of the shearer and analyzed the influence of the drum speed on the dynamic characteristics of the system.

Those previous studies only analyzed the dynamic characteristics of a single system or a single direction of the shearer. However, shearer is a large-scale and complex mechanical system, and the vibration characteristics of its various parts would interrelate each other. Under actual working condition consisting of joint development and uneven hardness distribution of coal rocks, strong impact load generated by the drum in cutting process would be transmit to the whole system of the shearer. The hydraulic tie bars are important parts used to connect the shearer body and the traction section. Its connection characteristics directly would affect the dynamic characteristics of the shearer.

Based on the above mentioned problems, this article comprehensively considered the connection characteristics of the shearer body with the traction part, the connection characteristics of the rocker arm with the traction part in existence of clearance, the support characteristics of the guide shoe and the smooth shoe, the intrinsic characteristics of the rocker arm, and the connection characteristics of the traction section with both walking box and support part. Based on theories of vibration mechanics and multi-body dynamics, a 13 DOF vertical–pitch coupling dynamics model of shearer was established. Corrected drum loads were proposed as the external excitation, the dynamic characteristics of key parts of the shearer were analyzed and finally experimental method was utilized to verify the accuracy of the established model and the solution results. The results of this research can provide a theoretical basis for automatic control, reliability study and fatigue life prediction of the key components of the shearer.

## Establishment of shearer dynamics model

### Establishment of vertical–pitch coupling dynamics model

**Figure 1:**
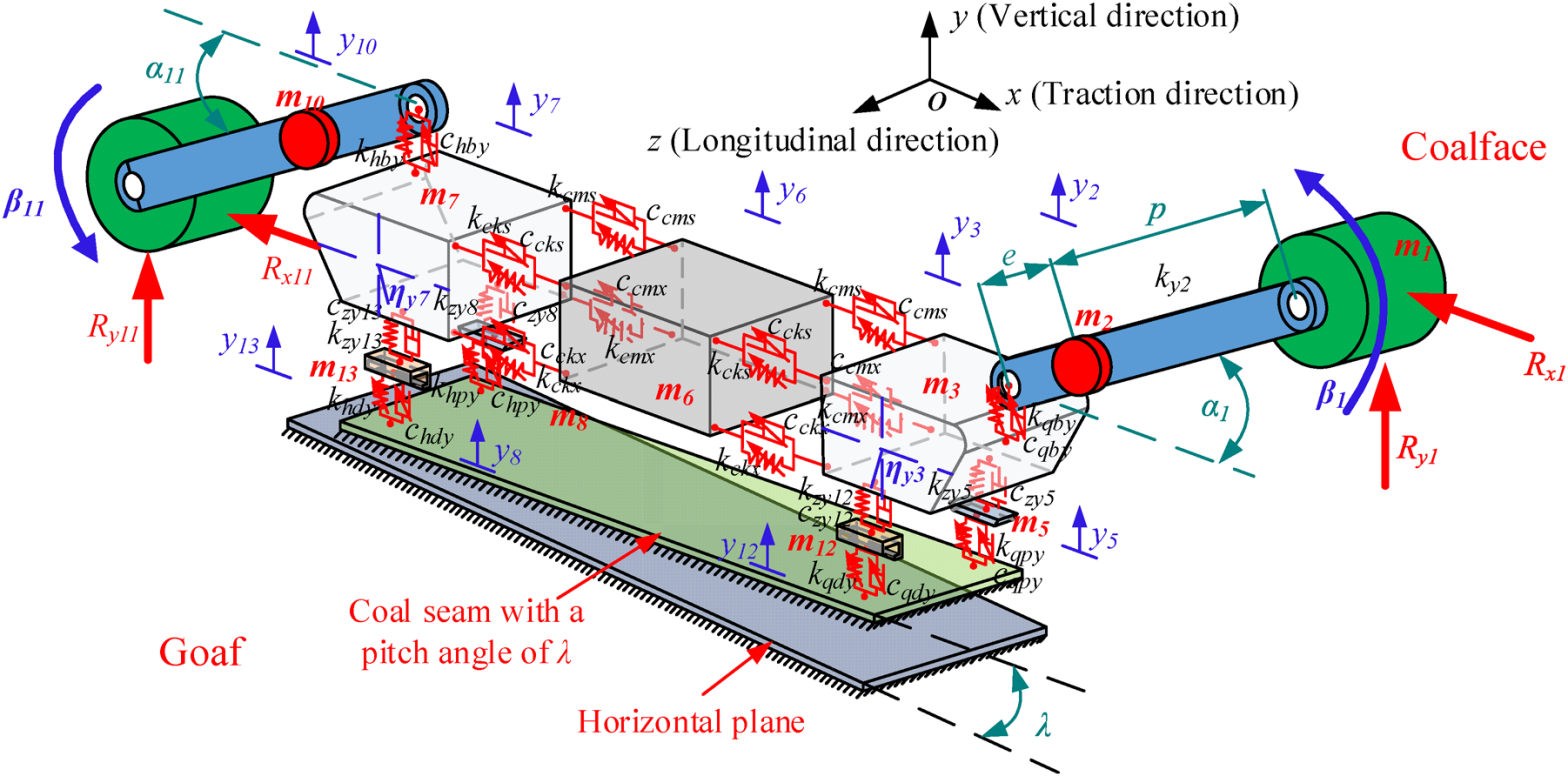
Vertical–pitch coupling nonlinear dynamics model of shearer.

Due to the complex structure of the shearer, the efficiency of the model solution and the sufficient accuracy of the vertical–pitch coupling dynamic characteristics are both considered during the model simplification. Based on the lumped parameter method, the shearer is divided into 11 parts, as shown in Fig. 1: front and rear drums, front and rear rocker arms, front and rear traction parts, front and rear walking boxes, front and rear support parts, and the shearer body. The cutting load of the drum is considered as the external excitation, the connection characteristics between parts and the support characteristics of the shearer are also taken into account. Moreover, those assumptions are included.

1. The quality of each part in the model is concentrated on the center of gravity.
2. The connection damping between parts in the model is viscous damping.
3. The front sections of the front and rear rocker arms are beams with no mass, which are connected to the front and rear drums, respectively.
4. The influence of the electrical system, hydraulic system and transmission system of the shearer on the dynamic characteristics of the overall machine is ignored.
5. The initial gaps on both sides of the connecting position of each part of the shearer are equal.

The description of symbols in Fig. 1 are shown in Table 1.

**Table 1:** Description of symbols in Fig. 1

Symbols	Names
m_1_, m_11_	The quality of the front and rear rollers, respectively
m_2_, m_10_	Mass of front and rear rocker arms, respectively
m_3_, m_7_	Quality of front and rear traction parts, respectively
m_12_, m_13_	The quality of the front and rear walking box, respectively
m_5_, m_8_	Mass of front and rear support, respectively
m_6_	The quality of the shearer body
*y*_*2*_, *y*_*10*_	Vibration displacement of front and rear rocker arms, respectively
*y*_*3*_, *y*_*7*_	Vibration displacement of front and rear traction parts, respectively
*y*_*5*_, *y*_*8*_	Vibration displacement of the front and rear supports, respectively
*y*_*12*_, *y*_*13*_	Vibration displacement of front and rear traveling boxes, respectively
*y*_*6*_	Vibration displacement of the shearer body
*k*_*y2*_, *k*_*y10*_	Equivalent stiffness of front and rear rocker arms, respectively
*k*_*qby*_, *c*_*qby*_ *k*_*hby*_, *c*_*hby*_	Connecting rigidity and connecting damping of front and rear rocker arms with the traction part
*k*_*zy5*_, *c*_*zy5*_ *k*_*zy8*_, *c*_*zy8*_	Connecting stiffness and damping of front and rear support parts with the traction part
*k*_*zy12*_, *c*_*zy12*_ *k*_*zy13*_, *c*_*zy13*_	Connecting stiffness and damping of front and rear walking box with the traction part
*k*_*qpy*_, *c*_*qpy*_ *k*_*hpy*_, *c*_*hpy*_	Support stiffness and damping of front and rear smooth shoes
*k*_*qdy*_, *c*_*qdy*_ *k*_*hdy*_, *c*_*hdy*_	Support rigidity and damping of front and rear guide shoes
*k*_*cms*_, *c*_*cms*_ *k*_*cmx*_, *c*_*cmx*_	Equivalent stiffness and damping of upper and lower hydraulic tie bars on the coal mining side
*k*_*cks*_, *c*_*cks*_ *k*_*ckx*_, *c*_*ckx*_	Equivalent stiffness and damping of upper and lower hydraulic tie bars on the goaf side
*α*_*1*_, *α*_*11*_	Lifting angle of front and rear rocker arms during working
*β*_*1*_, *β*_*11*_	Vibration swing angle of the front and rear rollers in *xoy* plane
*R*_*x1*_, *R*_*y1*_ *R*_*x11*_, *R*_*y11*_	Horizontal and vertical cutting load of front and rear drums
*η*_*y3*_, *η*_*y7*_	Vibration angle of the front and rear traction in *xoy* plane
*e*	Rotation radius of the center of gravity point for rocker arm
*p*	The distance between the roller's center of gravity point and the rocker's center of gravity point
*r*	Length of traction part
*a*	Distance between upper and lower hydraulic tie bars
*λ*	The pitch angle of the shearer

The kinetic energy, potential energy and dissipation energy of the system are analyzed as Eqs. (1–3), respectively:

1 $$ \begin{aligned} T & = T_{1} + T_{2} + T_{3} + T_{5} + T_{6} + T_{7} + T_{8} + T_{10} + T_{11} + T_{12} + T_{13} \\ & = \frac{1}{2}m_{1} \left[ {\left( {p \cdot \dot{\beta } \cdot \cos \alpha_{1} } \right)^{2} + \left( {\dot{y}_{2} - p \cdot \dot{\beta } \cdot \sin \alpha_{1} } \right)^{2} } \right] + \frac{1}{2}m_{2} \dot{y}_{2}^{2} + \frac{1}{2}m_{3} \dot{y}_{3}^{2} + \frac{1}{2}I_{y3} \cdot \dot{\eta }_{y3}^{2} + \frac{1}{2}m_{5} \dot{y}_{5}^{2} + \frac{1}{2}m_{6} \dot{y}_{6}^{2} \\ & \quad + \frac{1}{2}m_{7} \dot{y}_{7}^{2} + \frac{1}{2}I_{y7} \cdot \dot{\eta }_{y7}^{2} + \frac{1}{2}m_{8} \dot{y}_{8}^{2} + \frac{1}{2}m_{10} \dot{y}_{10}^{2} + \frac{1}{2}m_{11} \left[ {\left( {p \cdot \dot{\beta } \cdot \cos \alpha_{11} } \right)^{2} + \left( {\dot{y}_{10} - p \cdot \dot{\beta } \cdot \sin \alpha_{11} } \right)^{2} } \right] + \frac{1}{2}m_{12} \dot{y}_{12}^{2} + \frac{1}{2}m_{13} \dot{y}_{13}^{2} \\ \end{aligned} $$

2 $$ \begin{aligned} U & = \frac{1}{2}k_{y2} \left( {p \cdot \beta_{1} } \right)^{2} + \frac{1}{2}k_{qby} \left[ {y_{3} + \frac{1}{2}\left( {r \cdot \eta_{y3} - d_{y2} } \right) - y_{2} } \right]^{2} + \frac{1}{2}k_{zy5} \left( {y_{5} - y_{3} } \right)^{2} + \frac{1}{2}k_{qpy} y_{5}^{2} + \frac{1}{2}k_{zy12} \left( {y_{12} - y_{3} } \right)^{2} + \frac{1}{2}k_{qdy} y_{12}^{2} \\ & \quad + \frac{1}{2}k_{cms} \left[ {y_{3} + \frac{1}{2} \cdot l_{cms} \cdot \eta_{y3} + y_{7} + \frac{1}{2} \cdot l_{cms} \cdot \eta_{y7} - y_{6} } \right]^{2} + \frac{1}{2}k_{cmx} \left[ {y_{3} + \frac{1}{2} \cdot l_{cmx} \cdot \eta_{y3} + y_{7} + \frac{1}{2} \cdot l_{cmx} \cdot \eta_{y7} - y_{6} } \right]^{2} \\ & \quad + \frac{1}{2}k_{ckx} \left[ {y_{3} + \frac{1}{2} \cdot l_{ckx} \cdot \eta_{y3} + y_{7} + \frac{1}{2} \cdot l_{ckx} \cdot \eta_{y7} - y_{6} } \right]^{2} + \frac{1}{2}k_{cks} \left[ {y_{3} + \frac{1}{2} \cdot l_{cks} \cdot \eta_{y3} + y_{7} + \frac{1}{2} \cdot l_{cks} \cdot \eta_{y7} - y_{6} } \right]^{2} + \frac{1}{2}k_{hdy} y_{13}^{2} \\ & \quad + \frac{1}{2}k_{zy13} \left( {y_{13} - y_{7} } \right)^{2} + \frac{1}{2}k_{hpy} y_{8}^{2} + \frac{1}{2}k_{zy8} \left( {y_{8} - y_{7} } \right)^{2} + \frac{1}{2}k_{hby} \left[ {y_{7} + \frac{1}{2}\left( {r \cdot \eta_{y7} - d_{y10} } \right) - y_{10} } \right]^{2} + \frac{1}{2}k_{y10} \left( {p \cdot \beta_{11} } \right)^{2} \\ \end{aligned} $$

3 $$ \begin{aligned} D & = \frac{1}{2}c_{qby} \left( {\dot{y}_{3} + \frac{1}{2} \cdot r \cdot \dot{\eta }_{y3} - \dot{y}_{2} } \right)^{2} + \frac{1}{2}c_{zy5} \left( {\dot{y}_{5} - \dot{y}_{3} } \right)^{2} + \frac{1}{2}c_{qpy} \dot{y}_{5}^{2} + \frac{1}{2}c_{zy12} \left( {\dot{y}_{12} - \dot{y}_{3} } \right)^{2} + \frac{1}{2}c_{qdy} \dot{y}_{12}^{2} \\ & \quad + \frac{1}{2}c_{cms} \left[ {\dot{y}_{3} + \frac{1}{2} \cdot l_{cms} \cdot \dot{\eta }_{y3} + \dot{y}_{7} + \frac{1}{2} \cdot l_{cms} \cdot \dot{\eta }_{y7} - \dot{y}_{6} } \right]^{2} + \frac{1}{2}c_{cmx} \left[ {\dot{y}_{3} + \frac{1}{2} \cdot l_{cmx} \cdot \dot{\eta }_{y3} + \dot{y}_{7} + \frac{1}{2} \cdot l_{cmx} \cdot \dot{\eta }_{y7} - \dot{y}_{6} } \right]^{2} \\ & \quad + \frac{1}{2}c_{ckx} \left[ {\dot{y}_{3} + \frac{1}{2} \cdot l_{ckx} \cdot \dot{\eta }_{y3} + \dot{y}_{7} + \frac{1}{2} \cdot l_{ckx} \cdot \dot{\eta }_{y7} - \dot{y}_{6} } \right]^{2} + \frac{1}{2}c_{cks} \left[ {\dot{y}_{3} + \frac{1}{2} \cdot l_{cks} \cdot \dot{\eta }_{y3} + \dot{y}_{7} + \frac{1}{2} \cdot l_{cks} \cdot \dot{\eta }_{y7} - \dot{y}_{6} } \right]^{2} \\ & \quad + \frac{1}{2}c_{hdy} \dot{y}_{13}^{2} + \frac{1}{2}c_{zy13} \left( {\dot{y}_{13} - \dot{y}_{7} } \right)^{2} + \frac{1}{2}c_{hpy} \dot{y}_{8}^{2} + \frac{1}{2}c_{zy8} \left( {\dot{y}_{8} - \dot{y}_{7} } \right)^{2} + \frac{1}{2}c_{hby} \left( {\dot{y}_{7} + \frac{1}{2} \cdot r \cdot \dot{\eta }_{y7} - \dot{y}_{10} } \right)^{2} \\ \end{aligned} $$

where *l*_cms_ is the length of the hydraulic tie bar on the top of the mining side of the shearer, *l*_cmx_ is the length of the hydraulic tie bar at the bottom of the mining side of the shearer, *l*_cks_ is the length of the hydraulic tie bar on the top of the goaf side of the shearer, *l*_ckx_ is the length of the hydraulic tie rod at the bottom of the goaf side of the shearer and *d*_y2_ and *d*_y10_ are the connection clearances between the front and rear rocker arms with the traction part, respectively.

4 $$ {\mathbf{M}}\ddot{\mathbf{Y}} + {\mathbf{C}}\dot{\mathbf{Y}} + {\mathbf{KY}} = {\mathbf{F}} $$

Substituting Eqs. (1)–(3) into the Lagrange kinetic equations, the integral equation is processed into the form of Eq. (4) to obtain the matrices M, C, K and F (see “ESM Appendix”).

### Establishment of the stiffness model for key components

#### Connection stiffness model of the shearer body with traction part

According to the structure of the shearer, the stiffness models of the four hydraulic tie bars can be used to describe the connection state between the shearer body and the traction part. The relevant parameters of the hydraulic tie bars are shown in Table 2.

**Table 2:** Related parameters of hydraulic pull bar.

Codes of hydraulic tie bars	Position	Clamping length (mm)	Preload (mm)
*l*_*ckx*_	Bottom of goaf side	3460	6
*l*_*cks*_	Top of goaf side	4370	7
*l*_*cmx*_	Bottom of minging side	3460	6
*l*_*cms*_	Top of minging side	6080	10

The equivalent connection stiffness of the traction part with shearer body are:

5 $$ k_{cms} = \left\{ {\begin{array}{*{20}l} {\frac{{3E_{e} I_{e} }}{{l_{cms}^{3} }}} \hfill & {y_{3} + y_{7} + \frac{a}{2} \cdot \left( {\eta_{y3} + \eta_{y7} } \right) - y_{6} \ne 10} \hfill \\ 0 \hfill & {y_{3} + y_{7} + \frac{a}{2} \cdot \left( {\eta_{y3} + \eta_{y7} } \right) - y_{6} = 10} \hfill \\ \end{array} } \right. $$

6 $$ k_{cmx} = \left\{ {\begin{array}{*{20}l} {\frac{{3E_{e} I_{e} }}{{l_{cmx}^{3} }}} \hfill & {y_{3} + y_{7} + \frac{a}{2} \cdot \left( {\eta_{y3} + \eta_{y7} } \right) - y_{6} \ne 6} \hfill \\ 0 \hfill & {y_{3} + y_{7} + \frac{a}{2} \cdot \left( {\eta_{y3} + \eta_{y7} } \right) - y_{6} = 6} \hfill \\ \end{array} } \right. $$

7 $$ k_{cks} = \left\{ {\begin{array}{*{20}l} {\frac{{3E_{e} I_{e} }}{{l_{cks}^{3} }}} \hfill & {y_{3} + y_{7} + \frac{a}{2} \cdot \left( {\eta_{y3} + \eta_{y7} } \right) - y_{6} \ne 7} \hfill \\ 0 \hfill & {y_{3} + y_{7} + \frac{a}{2} \cdot \left( {\eta_{y3} + \eta_{y7} } \right) - y_{6} = 7} \hfill \\ \end{array} } \right. $$

8 $$ k_{ckx} = \left\{ {\begin{array}{*{20}l} {\frac{{3E_{e} I_{e} }}{{l_{ckx}^{3} }}} \hfill & {y_{3} + y_{7} + \frac{a}{2} \cdot \left( {\eta_{y3} + \eta_{y7} } \right) - y_{6} \ne 6} \hfill \\ 0 \hfill & {y_{3} + y_{7} + \frac{a}{2} \cdot \left( {\eta_{y3} + \eta_{y7} } \right) - y_{6} = 6} \hfill \\ \end{array} } \right. $$

#### Connection stiffness model of traction part with rocker arm in the existence of gap

**Figure 2:**
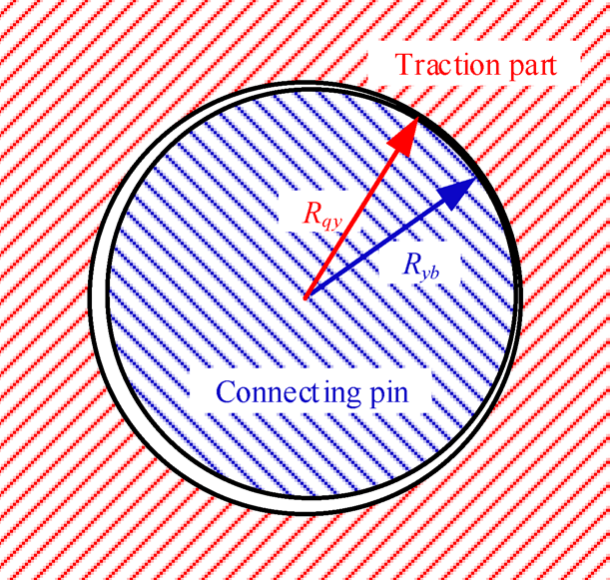
Schematic diagram of rocker arm pin connection.

Due to actual design size and long-term wear state, there is a certain clearance between the connecting pin of rocker arm and traction part, which affects the connection characteristics between rocker arm and traction part. In this research, it is assumed that the pin shaft and the rocker arm are regarded as rigid bodies, and the connecting clearance is equivalently compensated to the connection clearance between the traction part and the pin shaft. The connection diagram is shown in Fig. 2.

The size of the connection clearance is presented as Eq. (9):

9 $$ d_{yi} = 2 \cdot \left( {R_{qy} - R_{yb} } \right)\quad (i = 2,10) $$

where *d*_yi_ is the connection clearance between the pin of rocker arm and the traction part, *R*_qy_ is the radius of the connecting hole of traction part and *R*_yb_ is the radius of the connecting pin of rocker arm.

Under actual working conditions, the contact load between traction part and the connecting pin of rocker arm is constantly changing. Due to the connection clearance, the connection characteristics should be affected. Based on contact elasticity model, the slope nearby a certain instantaneous collision point in load vs. displacement curve that pertains to articulation mechanism with clearance is solved. The traction part is considered as rigid body. The connecting pin of rocker arm is considered as elastic body and its deformation is elastic. Therefore, the nonlinear contact stiffness coefficient between the traction part and the connecting pin of the rocker arm can be obtained as^18,19^:

10 $$ K_{yq} = \frac{1}{8}\pi E_{yq} \sqrt {\frac{{2 \cdot \delta_{yq} \left[ {3 \cdot \left( {R_{qy} - R_{yb} } \right) + 2 \cdot \delta_{yq} } \right]^{2} }}{{\left( {R_{qy} - R_{yb} + \delta_{yq} } \right)^{3} }}} $$

11 $$ E_{yq} = \left( {\frac{{1 - \upsilon_{y}^{2} }}{{E_{y} }} + \frac{{1 - \upsilon_{q}^{2} }}{{E_{q} }}} \right)^{ - 1} $$

where *E*_yq_ is the composite elastic modulus of the traction part and the connecting pin of rocker arm, *δ*_yq_ is the deformation of the connecting pin, *E*_y_ and *E*_q_ are elastic modulus values of the connecting pin and traction part, respectively, and *υ*_y_ and *υ*_q_ are the Poisson ratios of the connecting pin and the traction part, respectively.

At the initial position, the circle center points of the connecting hole of traction part and the connecting pin of rocker arm are assumed to be coincident, and thus the deformation of the connecting pin of shearer sliding shoe (*δ*_yq_) can be expressed as:

12 $$ \delta_{yq} = \left\{ {\begin{array}{*{20}l} {y_{j} + \eta_{yj} \cdot \frac{r}{2} - y_{i} - \frac{{d_{yi} }}{2}} \hfill & {y_{i} - \left( {y_{j} + \eta_{yj} \cdot \frac{r}{2}} \right) \le - \frac{{d_{yi} }}{2}} \hfill \\ 0 \hfill & { - \frac{{d_{yi} }}{2} < y_{i} - \left( {y_{j} + \eta_{yj} \cdot \frac{r}{2}} \right) \le \frac{{d_{yi} }}{2}} \hfill \\ {y_{i} - \left( {y_{j} + \eta_{yj} \cdot \frac{r}{2}} \right) - \frac{{d_{yi} }}{2}} \hfill & {y_{i} - \left( {y_{j} + \eta_{yj} \cdot \frac{r}{2}} \right) > \frac{{d_{yi} }}{2}} \hfill \\ \end{array} } \right.\quad \left( {\left\{ {\begin{array}{*{20}l} {i = 2} \hfill \\ {j = 3} \hfill \\ \end{array} } \right.\left\{ {\begin{array}{*{20}l} {i = 10} \hfill \\ {j = 7} \hfill \\ \end{array} } \right.} \right) $$

From Eqs. (10) and (12), the connection stiffness values between traction part and the front or rear rocker arms can be derived as:

13 $$ k_{qby} = \left\{ {\begin{array}{*{20}l} {\frac{1}{8}\pi E_{yq} \sqrt {\frac{{2 \cdot \left( {y_{3} + \eta_{y3} \cdot \frac{r}{2} - y_{2} - \frac{{d_{y2} }}{2}} \right) \cdot \left[ {3 \cdot \left( {R_{qy} - R_{yb} } \right) + 2 \cdot \left( {y_{3} + \eta_{y3} \cdot \frac{r}{2} - y_{2} - \frac{{d_{y2} }}{2}} \right)} \right]^{2} }}{{\left( {R_{qy} - R_{yb} + y_{3} + \eta_{y3} \cdot \frac{r}{2} - y_{2} - \frac{{d_{y2} }}{2}} \right)^{3} }}} } \hfill & {y_{2} - \left( {y_{3} + \eta_{y3} \cdot \frac{r}{2}} \right) \le - \frac{{d_{y2} }}{2}} \hfill \\ 0 \hfill & { - \frac{{d_{y2} }}{2} < y_{2} - \left( {y_{3} + \eta_{y3} \cdot \frac{r}{2}} \right) \le \frac{{d_{y2} }}{2}} \hfill \\ {\frac{1}{8}\pi E_{yq} \sqrt {\frac{{2 \cdot \left[ {y_{2} - \left( {y_{3} + \eta_{y3} \cdot \frac{r}{2}} \right) - \frac{{d_{y2} }}{2}} \right] \cdot \left[ {3 \cdot \left( {R_{qy} - R_{yb} } \right) + 2 \cdot \left( {y_{2} - \left( {y_{3} + \eta_{y3} \cdot \frac{r}{2}} \right) - \frac{{d_{y2} }}{2}} \right)} \right]^{2} }}{{\left( {R_{qy} - R_{yb} + y_{2} - \left( {y_{3} + \eta_{y3} \cdot \frac{r}{2}} \right) - \frac{{d_{y2} }}{2}} \right)^{3} }}} } \hfill & {y_{2} - \left( {y_{3} + \eta_{y3} \cdot \frac{r}{2}} \right) > \frac{{d_{y2} }}{2}} \hfill \\ \end{array} } \right. $$

14 $$ k_{hby} = \left\{ {\begin{array}{*{20}l} {\frac{1}{8}\pi E_{yq} \sqrt {\frac{{2 \cdot \left( {y_{3} + \eta_{y7} \cdot \frac{r}{2} - y_{10} - \frac{{d_{y10} }}{2}} \right) \cdot \left[ {3 \cdot \left( {R_{qy} - R_{yb} } \right) + 2 \cdot \left( {y_{7} + \eta_{y7} \cdot \frac{r}{2} - y_{10} - \frac{{d_{y10} }}{2}} \right)} \right]^{2} }}{{\left( {R_{qy} - R_{yb} + y_{7} + \eta_{y7} \cdot \frac{r}{2} - y_{10} - \frac{{d_{y10} }}{2}} \right)^{3} }}} } \hfill & {y_{10} - \left( {y_{7} + \eta_{y7} \cdot \frac{r}{2}} \right) \le - \frac{{d_{y10} }}{2}} \hfill \\ 0 \hfill & { - \frac{{d_{y10} }}{2} < y_{10} - \left( {y_{7} + \eta_{y7} \cdot \frac{r}{2}} \right) \le \frac{{d_{y10} }}{2}} \hfill \\ {\frac{1}{8}\pi E_{yq} \sqrt {\frac{{2 \cdot \left[ {y_{10} - \left( {y_{7} + \eta_{y7} \cdot \frac{r}{2}} \right) - \frac{{d_{y10} }}{2}} \right] \cdot \left[ {3 \cdot \left( {R_{qy} - R_{yb} } \right) + 2 \cdot \left( {y_{10} - \left( {y_{7} + \eta_{y7} \cdot \frac{r}{2}} \right) - \frac{{d_{y10} }}{2}} \right)} \right]^{2} }}{{\left( {R_{qy} - R_{yb} + y_{10} - \left( {y_{7} + \eta_{y7} \cdot \frac{r}{2}} \right) - \frac{{d_{y10} }}{2}} \right)^{3} }}} } \hfill & {y_{10} - \left( {y_{7} + \eta_{y7} \cdot \frac{r}{2}} \right) > \frac{{d_{y10} }}{2}} \hfill \\ \end{array} } \right. $$

#### Support stiffness models of both guide shoe and smooth shoe

In actual engineering, the contact surface cannot be absolutely smooth, which directly affects the dynamic characteristics of the mechanical system. The support characteristics of both guide shoe and smooth shoe are described according to the method^8^ that described the tangential contact characteristics on the joint surface between smooth shoe and middle groove.

**Figure 3:**
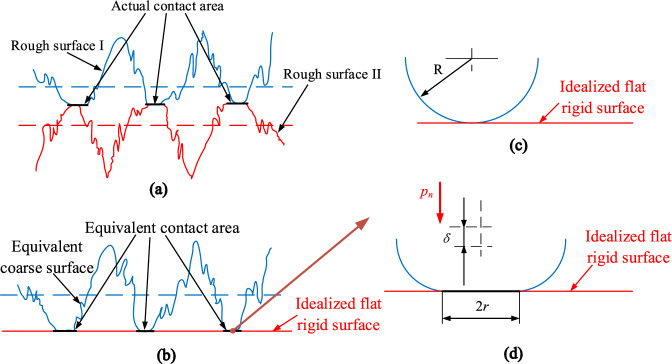
Schematic diagram of contact interfaces: (**a**) actual contact interface; (**b**) equivalent contact interface; (**c**) equivalent contact interface between a sphere asperity and a smooth surface, without zero applied load; (**d**) equivalent contact interface between a sphere asperity and a smooth surface, without applied load of *p*_n_.

Figure 3 are schematic diagrams of the actual contact model on the rough interface between guide shoe and pin row, and the rough interface between smooth shoe and middle groove. Figure 3a is the schematic diagram of the actual contact model on the rough interface. Based on GW model^20^ and CEB model^21^, the contact problem can be considered as equivalent to a rough surface contacting with an ideal smooth surface, where a single asperity in the equivalent contact area is regarded as a sphere with an equivalent curvature radius *R*, as shown in Fig. 3b. As no load applied, the contact state between the asperity and the smooth surface is presented, as seen in Fig. 3c. As a normal load *p*_n_ applied, the contact state is presented in Fig. 3d, where *δ* is the normal deformation of the equivalent sphere, and *r* is the contact radius of the equivalent sphere.

According to the Ref.^22^, the modeling method of rough surface for elastic contact mechanics model, the equations of support stiffness on front and rear guiding shoes and front and rear smooth shoes are presented as Eqs. (15–18), respectively:

15 $$ k_{qdy} = \frac{2D}{{\sqrt \pi \left( {1 - D} \right)\left( {\frac{{1 - \upsilon_{d}^{2} }}{{E_{d} }} + \frac{{1 - \upsilon_{x}^{2} }}{{E_{x} }}} \right)}}\psi^{{\left( {2 - D} \right)/2}} \cdot y_{12}^{D/2} \cdot \left[ {y_{12}^{{\left( {1 - D} \right)/2}} - \left( {\frac{{y_{\mu xd} }}{2}} \right)^{{\left( {1 - D} \right)/2}} } \right] $$

16 $$ k_{hdy} = \frac{2D}{{\sqrt \pi \left( {1 - D} \right)\left( {\frac{{1 - \upsilon_{d}^{2} }}{{E_{d} }} + \frac{{1 - \upsilon_{x}^{2} }}{{E_{x} }}} \right)}}\psi^{{\left( {2 - D} \right)/2}} \cdot y_{13}^{D/2} \cdot \left[ {y_{13}^{{\left( {1 - D} \right)/2}} - \left( {\frac{{y_{\mu xd} }}{2}} \right)^{{\left( {1 - D} \right)/2}} } \right] $$

17 $$ k_{qpy} = \frac{2D}{{\sqrt \pi \left( {1 - D} \right)\left( {\frac{{1 - \upsilon_{p}^{2} }}{{E_{p} }} + \frac{{1 - \upsilon_{z}^{2} }}{{E_{z} }}} \right)}}\psi^{{\left( {2 - D} \right)/2}} \cdot y_{5}^{D/2} \cdot \left[ {y_{5}^{{\left( {1 - D} \right)/2}} - \left( {\frac{{y_{\mu pz} }}{2}} \right)^{{\left( {1 - D} \right)/2}} } \right] $$

18 $$ k_{hpy} = \frac{2D}{{\sqrt \pi \left( {1 - D} \right)\left( {\frac{{1 - \upsilon_{p}^{2} }}{{E_{p} }} + \frac{{1 - \upsilon_{z}^{2} }}{{E_{z} }}} \right)}}\psi^{{\left( {2 - D} \right)/2}} \cdot y_{8}^{D/2} \cdot \left[ {y_{8}^{{\left( {1 - D} \right)/2}} - \left( {\frac{{y_{\mu pz} }}{2}} \right)^{{\left( {1 - D} \right)/2}} } \right] $$

The symbols of Eqs. (15–18) are shown in Table 3.

**Table 3:** Symbol description of Eqs. (15–18).

Symbols	Name
*E*_*d*_	Elastic modulus of guide shoe
*E*_*x*_	Elastic modulus of pin row
*E*_*p*_	Elastic modulus of smooth shoe
*E*_*z*_	Elastic modulus of the middle groove
*υ*_*d*_	Poisson's ratio of guided shoes
*υ*_*x*_	Poisson's ratio of pin row
*υ*_*p*_	Poisson's ratio of smooth boots
*υ*_*z*_	Poisson's ratio in the middle trough
*D*	Fractal dimension of joint surface
*ψ*	The expansion factor of the contact size distribution of asperities (ψ > 1), which is related to the fractal dimension *D* [22]
*y*_*μxd*_	Critical contact cross-sectional area of elastic–plastic deformation between guide shoe and pin rail asperity
*y*_*μpz*_	Critical contact cross-sectional area of elastic–plastic deformation between smooth shoe and middle groove asperity

#### Stiffness model of the rocker arm

Combined with the shearer rocker arm stiffness model in Ref.^23^ and the intrinsic characteristics of the shearer rocker arm in this study, the equivalent stiffness of the rocker arm can be derived as

19 $$ k_{y2} = k_{y10} = \frac{{3E_{e} I_{e} }}{{p^{3} }} $$

where *E*_e_ is the elastic modulus for the material of the shearer rocker arm and *I*_e_ is the moment of inertia for the cross section of the shearer rocker arm.

## Load model of the shearer drum and experimental research

### The load correction model of the shearer drum

The load determination of the shearer drum is the prerequisite for the analysis of the dynamic characteristics of the whole machine. Taking into account the stability of the solution process of the shearer dynamics system and the accuracy of the solution result, the shearer traction speed correction coefficient is introduced to the correction on the traditional load model of the drum^24^. Thus, the load model of the shearer drum with corrected traction speed can be established as follows:

20 $$ \left\{ {\begin{array}{*{20}l} {R_{gx} = k_{vx} \cdot \sum\limits_{i = 1}^{{N_{c} }} {\left( {Z_{i} \cos \phi_{i} + Y_{i} \sin \phi_{i} } \right)} } \hfill \\ {R_{gy} = k_{vy} \cdot \sum\limits_{i = 1}^{{N_{c} }} {\left( { - Z_{i} \sin \phi_{i} + Y_{i} \cos \phi_{i} } \right)} } \hfill \\ {R_{gz} = k_{vz} \cdot \sum\limits_{i = 1}^{{N_{c} }} {\left( {X_{i} } \right)}  } \hfill \\ \end{array} } \right. $$

where *R*_gx_, *R*_gy_ and *R*_gz_ are the cutting load of the drum in traction direction, vertical direction, and axial direction, respectively; *N*_c_ is the total amount of cutting teeth of the drum participating in cutting; *R*_g_ is the radius of the drum; *φ*_i_ is the included angle between the central axis of the i-th pick and the vertical direction of the drum; *X*_i_ is the lateral resistant force of the i-th pick participating in cutting; *Y*_i_ is the traction resistant force of the i-th pick participating in cutting; *Z*_i_ is the cutting resistant force of the i-th pick participating in cutting; *k*_vx_, *k*_vy_, and *k*_vz_ are the traction speed correction coefficients.

### Determination of the correction coefficient

**Figure 4:**
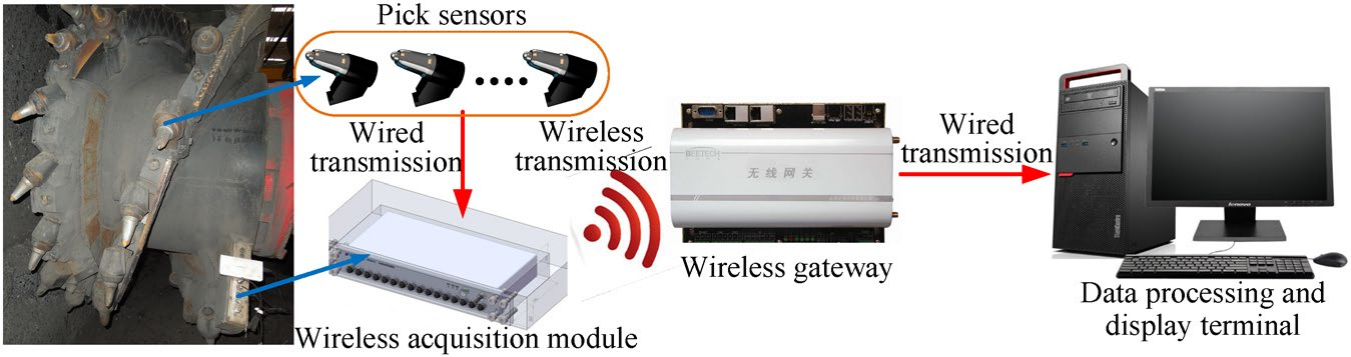
Data acquisition system of picks.

The experimental method is applied to determine the traction speed correction coefficient in the drum load model. Taking into account the limitations of the experimental conditions in underground coal mine, using the mechanical testing and analysis experimental platform of at National Energy Mining Equipment R&D Center (Zhangjiakou, China), cutting experiments with various shearer traction speeds (1.5 m/min, 2 m/min, 2.5 m/min, 3 m/min and 3.5 m/min) are complemented. During the experiment, nine pick sensors are installed on the spiral blade of the drum, as shown in Fig. 4.

Those sensors are connected to the wireless acquisition module installed at the end of the spiral blade of the drum, in order to transmit collected data from sensors to the wireless acquisition module. Then, the data is transmitted to the data collection terminal through the wireless gateway by means of wireless communication. The three-directional forces of the drum are then calculated and obtained. Table 4 presents the average values of the three-directional cutting loads under different traction directions. The traditional calculated values are calculated by the traditional drum load formula, and the experimental values are obtained via the drum load experiment.

**Table 4:** Experimental values (EV) and traditional calculated values (TCV) of the drum.

Working condition	*R*_*gx*_/kN in traction direction			*R*_*gy*_/kN in vertical direction			*R*_*gz*_/kN in axis direction		
	EV	TCV	EV/TCV	EV	TCV	EV/TCV	EV	TCV	EV/TCV
***v *(m/min)**									
1.5	33.52	35.43	0.946	20.13	22.51	0.894	7.87	8.84	0.890
2	35.23	36.75	0.958	22.56	24.68	0.914	9.42	10.26	0.918
2.5	41.34	42.52	0.972	30.17	32.82	0.919	17.22	18.78	0.922
3	46.62	47.38	0.978	33.35	36.12	0.923	20.15	21.69	0.929
3.5	51.42	52.45	0.980	40.35	43.18	0.934	27.35	29.28	0.934

The traction speed correction coefficients of the three-directional cutting loads of the shearer drum are

21 $$ \left\{ \begin{gathered} k_{vx} = 0.932 + 0.018 \cdot v \hfill \\ k_{vy} = 0.872 + 0.018 \cdot v \hfill \\ k_{vz} = 0.869 + 0.020 \cdot v \hfill \\ \end{gathered} \right. $$

where *v* is the traction speed of the shearer.

When the shearer traction speed is 3 m/min, the corrected values of the three-directional loads of the drum are selected, and the corresponding traditional calculated values and experimental values are compared, as shown in Table 5. It can be seen that the corrected value of the three-way load of the drum is closer to the experimental value and the relative errors are 0.77%, 2.67%, and 1.59% respectively.

**Table 5:** Comparison of experimental values (EV) with corrected values (CV) for the three-directional loads of the drum.

Operation condition	*R*_*gx*_/kN in traction direction				*R*_*gy*_/kN in vertical direction				*R*_*gz*_/kN in axis direction			
	EV	CV	Error%	TCV	EV	CV	Error%	TCV	EV	CV	Error%	TCV
*v* = 3 m/min	46.62	46.98	0.77	47.38	33.35	34.23	2.67	36.12	20.15	20.47	1.59	21.69

## Numerical solution and analysis

The Newmark-β method is applied to solve the vertical–pitch coupling dynamics model of the shearer.

- Step 1: The mass matrix ***M***, stiffness matrix ***K***, and damping matrix ***C*** is determined.
- Step 2: The initial value is given as ${\mathbf{Y}}_{0} = 0$, ${\dot{\mathbf{Y}}}_{0} = 0$, ${\ddot{\mathbf{Y}}}_{0} = 0$.
- Step 3: $\Delta t$, $\delta$ and $\beta$ were set as:
  $$ a_{0} = \frac{1}{{\beta \Delta t^{2} }};a_{1} = \frac{\delta }{\beta \Delta t};a_{2} = \frac{1}{\beta \Delta t};a_{3} = \frac{1}{2\beta } - 1;a_{4} = \frac{\delta }{\beta } - 1;a_{5} = \frac{\delta }{2\beta } - 1;a_{6} = \Delta t\left( {1 - \delta } \right);a_{7} = \delta \Delta t; $$
- Step 4: $${\hat{\mathbf{K}}} = a_{0} {\mathbf{M}} + a_{1} {\mathbf{C}} + {\mathbf{K}}$$; $${\hat{\mathbf{K}}\mathbf{X}}_{t + \Delta t} = {\hat{\mathbf{F}}}_{t + \Delta t}$$.
- Step 5: $${\hat{\mathbf{F}}}_{t + \Delta t} = {\mathbf{F}}_{t + \Delta t} + {\mathbf{\rm M}}\left( {a_{0} {\mathbf{Y}}_{t} + a_{2} {\dot{\mathbf{Y}}}_{t} + a_{3} {\ddot{\mathbf{Y}}}_{t} } \right) + C\left( {a_{1} {\mathbf{Y}}_{t} + a_{4} {\dot{\mathbf{Y}}}_{t} + a_{5} {\ddot{\mathbf{Y}}}_{t} } \right)$$.
- Step 6: $${\mathbf{LDL}}^{T} {\mathbf{Y}}_{t + \Delta t} = {\hat{\mathbf{F}}}_{t + \Delta t} \Rightarrow {\mathbf{Y}}_{t + \Delta t}$$.
- Step 7: $${\ddot{\mathbf{Y}}}_{t + \Delta t} = a_{0} \left( {{\mathbf{Y}}_{t + \Delta t} - {\mathbf{Y}}_{t} } \right) - a_{2} {\dot{\mathbf{Y}}}_{t} - a_{3} {\ddot{\mathbf{Y}}}_{t}$$; $${\dot{\mathbf{Y}}}_{t + \Delta t} = {\dot{\mathbf{Y}}}_{t} + a_{6} {\ddot{\mathbf{Y}}}_{t} + a_{7} {\ddot{\mathbf{Y}}}_{t + \Delta t}$$.

According to the operating parameters with the best economic benefits obtained from the long-term work of the shearer in coal mine roadway, the shearer traction speed is set as 3 m/min, the cutting depth is 600 mm, the drum speed is 32 r/min, the pitch angle is 0, the lifting angle of the front rocker arm is 27°, the lifting angle of the rear rocker arm is − 15°, and the coal rock solidity coefficient is *f* = 3. The vibration response curves of each key part of the shearer were intercepted in 20 s.

### Characteristic analysis of vibration displacement and vibration swing angle

**Figure 5:**
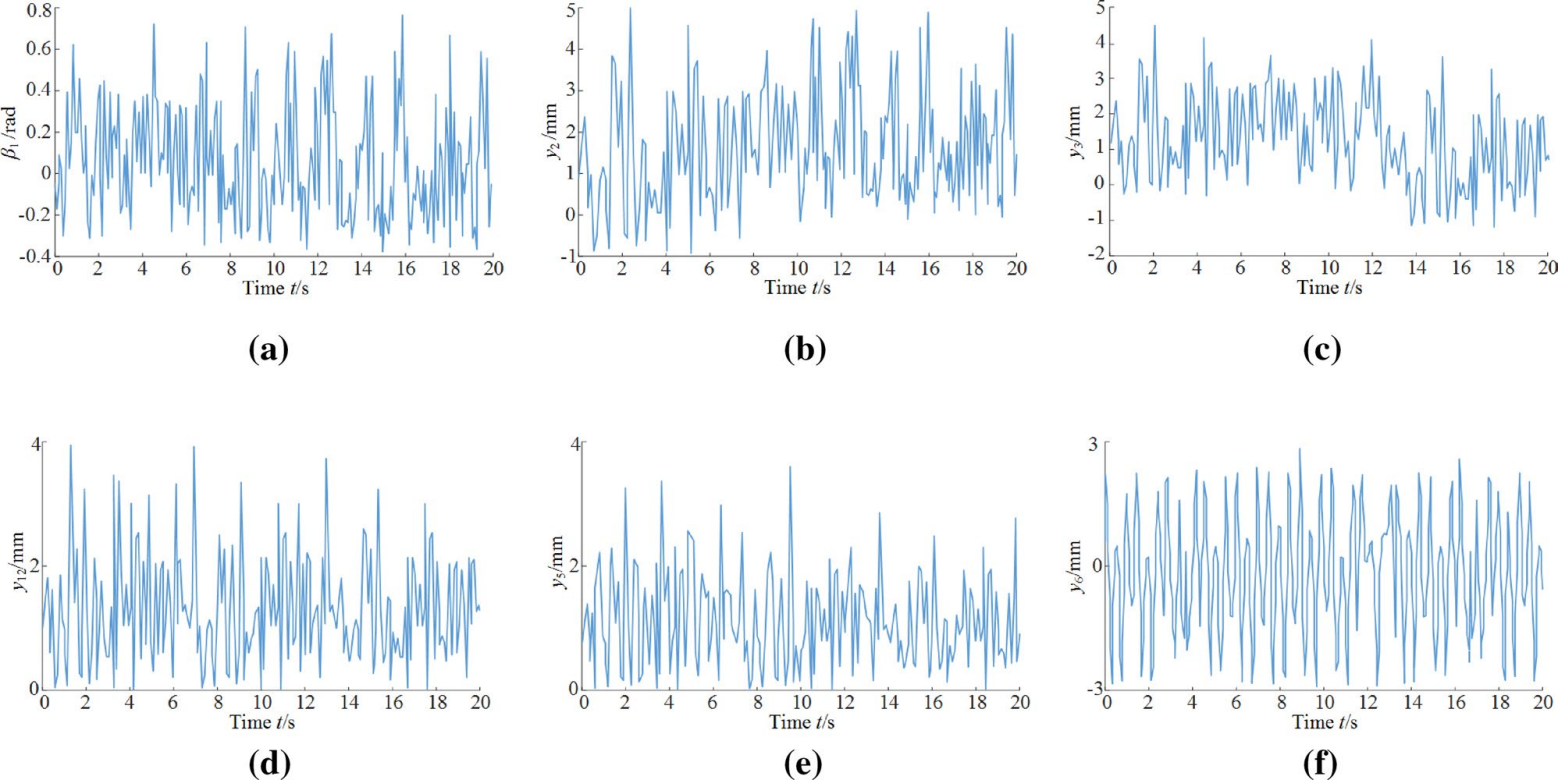
Vibration displacement (VD) and vibration swing angle (VSA) curves of the key parts of the shearer: (**a**) VSA of the front drum; (**b**) VD of front rocker arm; (**c**) VD of the front traction part; (**d**) VD of the front walking box; (**e**) VD of the front support part; (**f**) VD of the shearer body.

The coal miner drum is the external load input for the entire power system, the rocker arm is directly connected to the drum in the entire dynamics model, and the shearer cutting part holds a small mass and low inertia. As shown in Fig. 5, the vibration of the front cutting part has a large fluctuation range and is affected by the connection clearance between the rocker arm and the traction part. The vibration of the drum and the rocker arm turn to negative at certain moments. The vibration swing angle of the front drum fluctuate in the range of − 0.4 to 0.8 rad, and the vibration displacement of the front rocker arm fluctuate in the range of − 1 to 5 mm.

In the description process of the shearer dynamics model, it is assumed that the traction part is connected with both the walking box and the supporting part under fixed stiffness values, and the influence of the dynamic characteristics of the scraper conveyor on the shearer system is not considered. As seen in Fig. 5c–e, the fluctuation range and trend of the vibration displacements of traction part, walking box and support part are basically consistent with each other; affected by the unevenness of the joint surfaces of both the guide shoe and the smooth shoe, those vibration displacements fluctuate in a quite random manner and jump to a peak at a certain moment. Affected by the connection clearance between rocker arm and traction part, the vibration displacement of traction part abruptly change to − 1 mm at some moments, and the vibration displacements of the front traction part, front walking box and front support part fluctuate in the range of − 1 to 5 mm, − 1 to 4.5 mm, 0–4 mm and 0–4 mm, respectively.

### Characteristic analysis of vibration acceleration

Based on the above analysis, the vibration displacement trend of the shearer drum and rocker arm is similar, and the vibration displacement trend of the traction part, support part and the walking box is similar too. The rocker arm and the walking box are important parts for the walking and cutting process of the shearer. Their characteristics of connection with clearance and support directly affects the vibration connection speed curve. Therefore, the following content mainly analyzes the vibration acceleration characteristics of the front rocker arm and the front walking box of the shearer.

**Figure 6:**
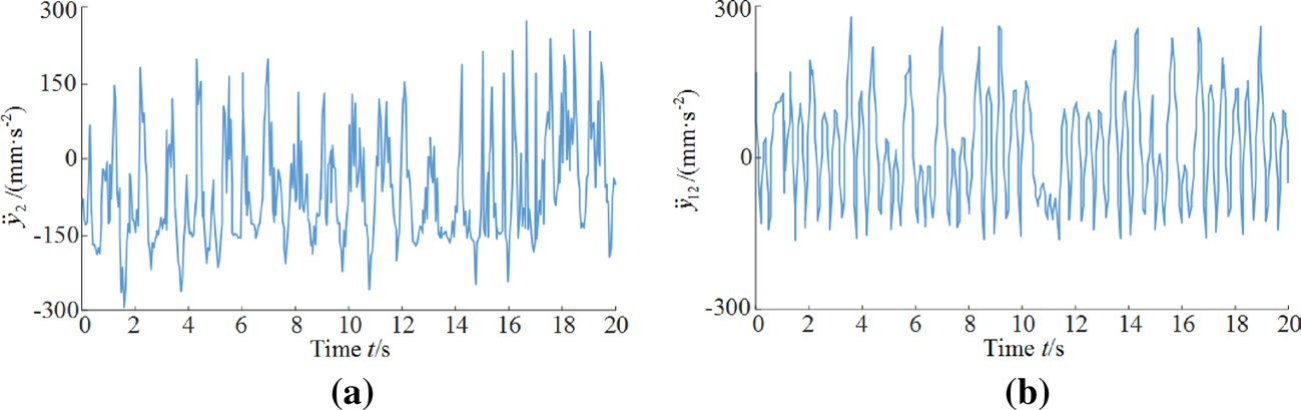
Vibration acceleration curve of the key parts of the shearer: (**a**) front rocker arm; (**b**) front walking box.

The rocker arm is subject to great load fluctuation since it is directly connected to the drum and thus indirectly affected by external excitation. It can be seen from Fig. 6a that vibration acceleration of the rocker arm mainly fluctuate from − 200 to 200 mm/s^2^. The vibration acceleration curve of the rocker arm, affected by the characteristics of the clearance connection to the traction part, performs very unstable. The vibration acceleration experience sudden jump to ~ ± 300 mm/s^2^ at certain moments, representing large impact loads.

It can be seen from Fig. 6b that the vibration acceleration of the front walking box mainly fluctuates from − 150 to 150 mm/s^2^. At certain moments, the vibration acceleration curve of the walking box experiences sudden jump to 300 mm/s^2^, representing the emergence of large impact load, owing to the unevenness of the joint surface between guide shoe and pin rail.

### Characteristic analysis of frequency domain response

**Figure 7:**
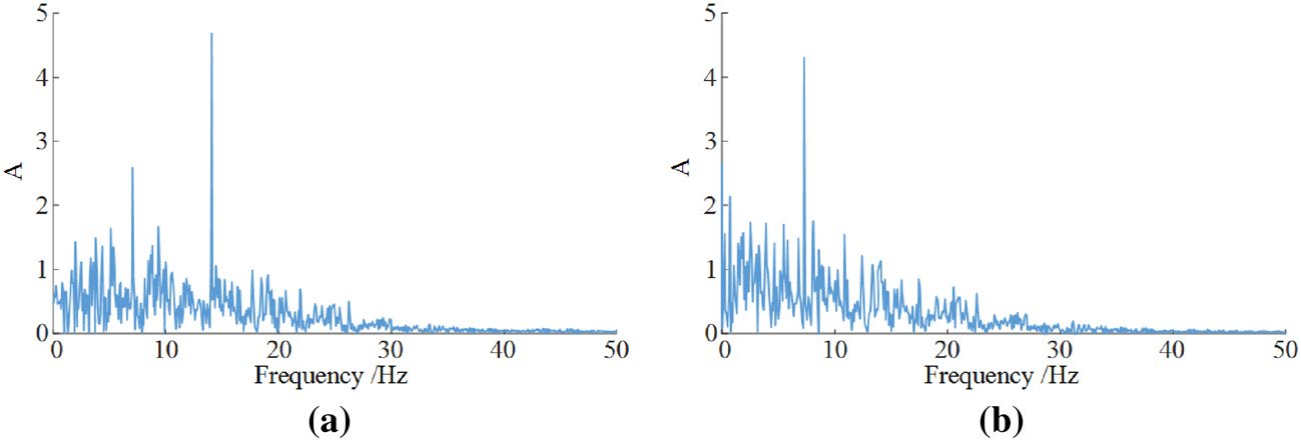
Vibration frequency spectra of the key parts of the shearer of (**a**) front rocker arm and (**b**) front walking box.

Due to the large mass of key components of the shearer, the response frequencies of every key components are low and had little distinction with each other. Figure 7 shows the frequency domain characteristic curves of front rocker arm and front walking box of the shearer. The frequency response spectra are both continuous. When the vibration frequency is 13.72 Hz, the vibration response of the front rocker arm reaches to the largest, and the vibration response of the front walking box is the largest when its vibration frequency is 7.98 Hz. Moreover, there are some lower frequency characteristics mixed in the vibration spectrum.

### Analysis of dynamic characteristics under different working conditions

**Figure 8:**
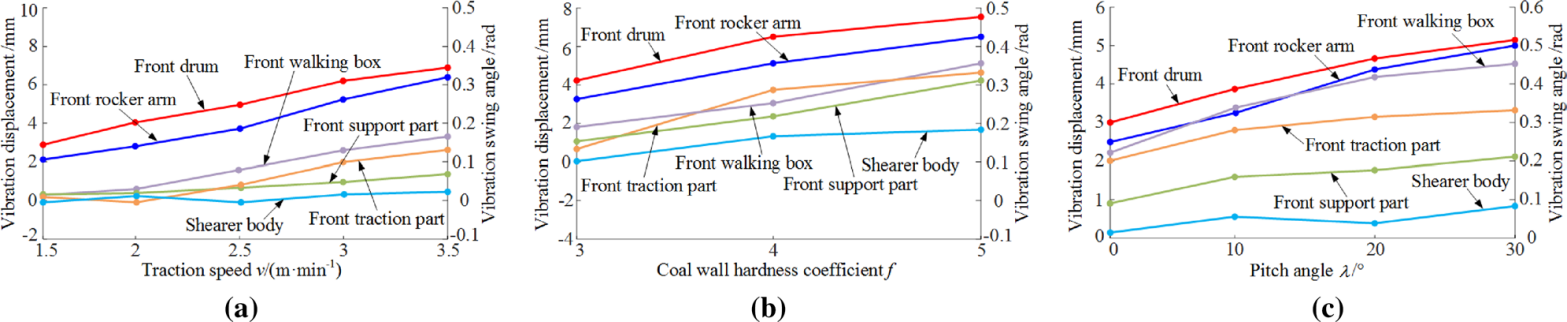
Vibration displacement of key parts of the shearer under different working conditions: under varied (**a**) traction speeds, (**b**) hardness values of coal rock and (**c**) pitch angles.

Based on the Newmark-β method and single variable method, the vibration displacement and vibration swing angle of key parts of the shearer under different working conditions are solved. The vibration displacement of rocker arm is converted into vibration swing angle according to its geometric relationship with drum, aiming to express the relationship between drum and rocker arm more intuitively. Then the average values of both vibration swing angles and vibration displacements for parts of the shearer are solved. Results under different working conditions are plotted in Fig. 8.

In Fig. 8a, the pitch angle of the shearer is 0, the coal wall hardness coefficient (*f*) is 3 and the traction speed varied from 1.5 to 3.5 m/min. As traction speed increases, the vibration swing angles of both front rocker arm and front drum obvious change with similar changing trend. When traction speed was 3.5 m/min, the average vibration swing angles of those two parts are approximately the same. The change range of vibration swing angle are 0.17–0.34 rad and 0.15–0.32 rad for front drum and front rocker arm, respectively. As traction speed increased from 2 to 3.5 m/min, the vibration displacements of front walking box and traction part obviously increase—from 0.30 to 2.37 mm for front drum and from 0.11 to 2.01 mm for front rocker arm.

In Fig. 8b, the traction speed of the shearer is 3 m/min, the pitch angle is 0 and the coal wall hardness coefficient (*f*) varies from 3 to 5. The average vibration displacements of every parts of the shearer increase as the increase of coal wall hardness. The vibration swing angles of front drum and front rocker arm have the same changing trend. As *f* increased from 3 to 4, the average vibration swing angles for those two parts increase at high level, from 0.32 to 0.48 rad for front drum and from 0.29 to 0.42 rad for front rocker arm. Affected by the vibration of rocker arm, the average vibration displacement of the traction part increases at a high rate when *f* is in the range of 3–4 and then experiences a relatively lower increase rate when *f* is in the range of 4–5. The corresponding vibration displacement ranges from 0.50 to 4.20 mm. The change trend of the vibration displacements of front walking box and support part are similar. The vibration displacements are from 1.96 to 4.64 mm for front walking box and from 1.00 to 4.00 mm for support part.

In Fig. 8c, the shearer traction speed is 3 m/min, the coal wall firmness coefficient *f* is 3 and the shearer pitch angle is 0°–30°. The vibration displacements and vibration swing angles of front drum, front rocker arm and front walking box increase at a relatively high rate. As pitch angle increases, the difference between vibration swing angles of drum and rocker arm diminishes. When the pitch angle reaches to 30°, the vibration swing angle of front drum and front rocker are 0.51 rad and 0.50 rad, respectively. As the pitch angle increases from 0° to 20°, the average vibration displacement of the front walking box increases from 2.20 to 4.1 mm, in a relatively higher rate than that of the other parts. As pitch angle increases from 0° to 10°, the average value of the vibration displacement of front traction part, front support part and fuselage increase significantly. As pitch angle increases from 10° to 20°, their increase rates slow down. As the increase of pitch angle, the vibration displacement of walking box monotonously increases from 2.29 to 4.32 mm and the swing angle of front rocker arm and rocker arm increase from 0.36 to 0.52 rad and from 0.27 to 0.50 rad, respectively.

## Experiment research on mechanical characteristics of the shearer

### Experiment platform

**Figure 9:**
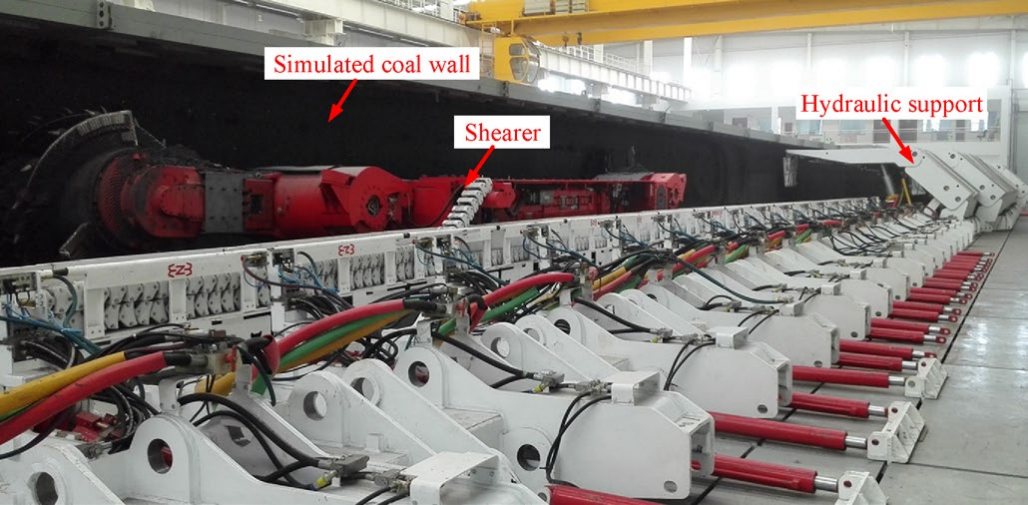
The mechanical testing and analysis experimental platform for fully mechanized working face.

In order to verify the correctness of the established dynamics model of the shearer and the accuracy of the corresponding solution results, the cutting and mechanical experiments are carried out using the mechanical testing and analysis experimental platform for fully mechanized working face at National Energy Mining Equipment R&D Center (Zhangjiakou, China). As shown in Fig. 9, the experimental platform is mainly composed of 1:1 simulated coal wall, a shearer, a scraper conveyor, hydraulic supports and data acquisition system. The model number of the shearer is MG500/1130WD.

In order to ensure the facticity and reliability of the data measured in the experiment, the simulated coal wall for the experiment should be able to simulate the geological structure and mechanical properties of the real coal seam as much as possible. As the largest supply base of high-quality thermal coal in China, the coal at coal mines from Datong in Shanxi province holds properties of well-developed joints, less impurities, high calorific value and high hardness, which are representative in China. Thus, the coal seam in that area is selected and used as the simulation object of the experimental coal wall. The main raw material of simulated coal wall is coal, supplemented by cement. The selected coal and cement are mixed through water, with the addition of an appropriate amount of water reducing agent^25^. The simulated coal wall is poured in the way of layer by layer. The thickness of a layer is 300 mm. The parameters of main raw materials are shown in Table 6. The total length, width and height of the simulated coal wall is 70 m, 4 m and 1.5 m, respectively. The hardness coefficient (*f*) of the front 35 m coal wall is 3, and that of the left 35 m coal wall is 4.

**Table 6:** Main raw material parameters of simulated coal wall.

Particle size of coal		Ordinary Portland cement	
Fine coal	Coarse coal	Strength grade	Margin coefficient
≤ 5 mm	5–50 mm	32.5	1.05

### Data collection and transmission method

**Figure 10:**
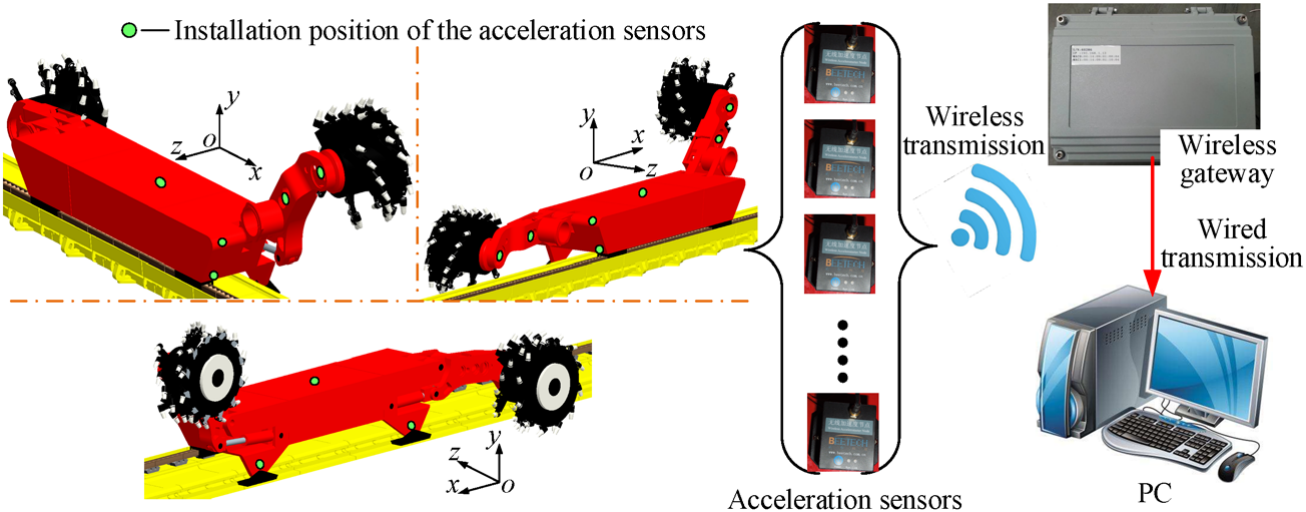
The data acquisition system of vibration acceleration for key parts of the shearer.

Taking into account the safety of the entire experimental process and the reliability of the data collection, a combination of wireless and wired data transmission is used in the entire experimental data collection process. As shown in Fig. 10, 13 wireless acceleration sensors are used to measure the vibration characteristics of front and rear drums, front and rear rocker arms, front and rear traction parts, front and rear walking boxes, front and rear supports and the fuselage of the shearer. Collected data are uploaded to the wireless gateway by means of wireless transmission, and the wireless gateway transmit the data to the terminal PC through wired transmission. Finally, the collected data are processed in the PC and displayed in the form of graphics.

**Figure 11:**
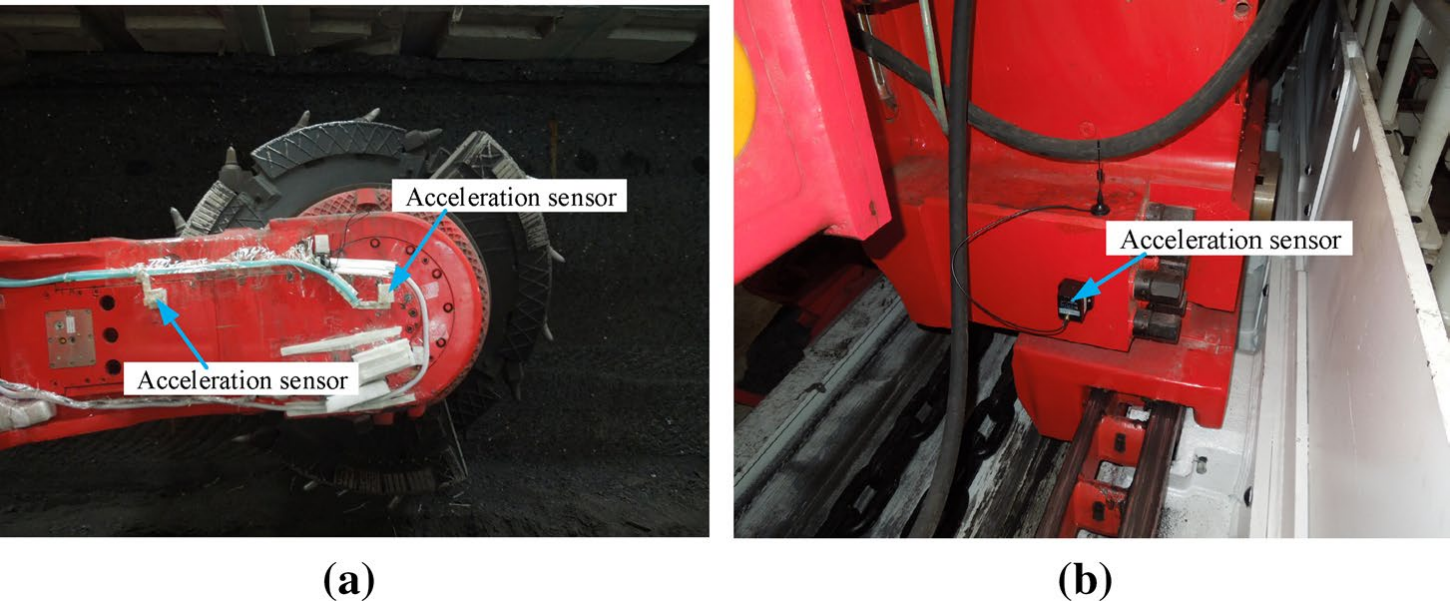
The installation position of the vibration acceleration sensor of the rocker arm and the walking box, for rocker arm (left) and drum (right) (**a**) and walking box (**b**).

Based on the above analysis, the installation positions of acceleration sensor for rocker arm and walking box are mainly introduced. As shown in Fig. 11a, the center of gravity position of the rocker arm is chosen to install the acceleration sensor. To avoid affection by coal falling, the sensor is properly packaged. As shown in Fig. 11b, to avoid the affection by coal falling, the collision of coal baffle of the scraper conveyor and the pin row, the end face of the walking box near the center of gravity are selected to install the acceleration sensors.

### Comparative analysis of theoretical and experimental research

The vibration acceleration curve and frequency domain response curve of the front rocker arm and walking box obtained by the simulation solution are compared with the experimental results. Among them, the working condition parameters are: traction speed of the shearer is 3 m/min, the coal wall hardness (*f*) coefficient is 3 and the mining pitch angle is 0.

**Figure 12:**
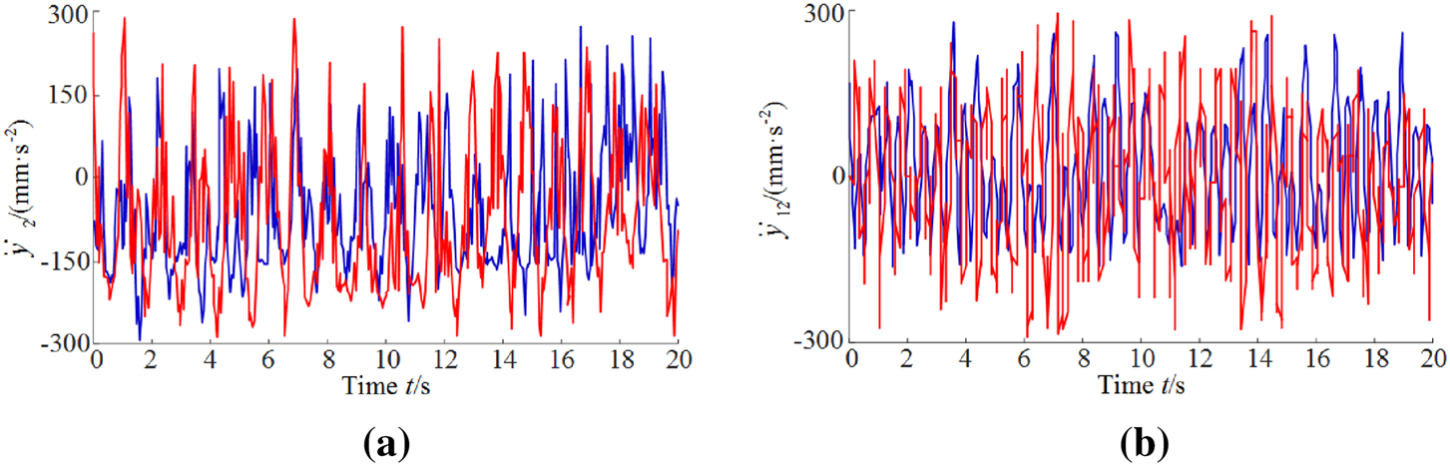
Vibration acceleration of rocker arm (**a**) and walking box (**b**).

As seen in Fig. 12, the vibration frequency from the simulation curves for rocker arm and walking box are basically consistent with those of the experimental ones. The vibration amplitudes from the experimental curves are larger. Since the polarity of vibration acceleration value indicates the direction, it can be seen from Table 7 that the average values of vibration acceleration from the experimental curves are greater than those from the simulation curve. The relative errors between the simulated and the experimental values for the rocker arm and the walking box are 8.70%, 6.91%, respectively.

**Table 7:** Comparison of simulated and experimental values.

	Rocker arm		Walking box	
	Vibration acceleration (rad/s^2^)	Dominant frequency (Hz)	Vibration acceleration (mm/s^2^)	Dominant frequency (Hz)
Simulated value	− 0.161	13.72	0.159	7.98
Experimental value	− 0.175	14.24	0.170	8.76
Relative error (%)	8.70	3.79	6.91	9.77

**Figure 13:**
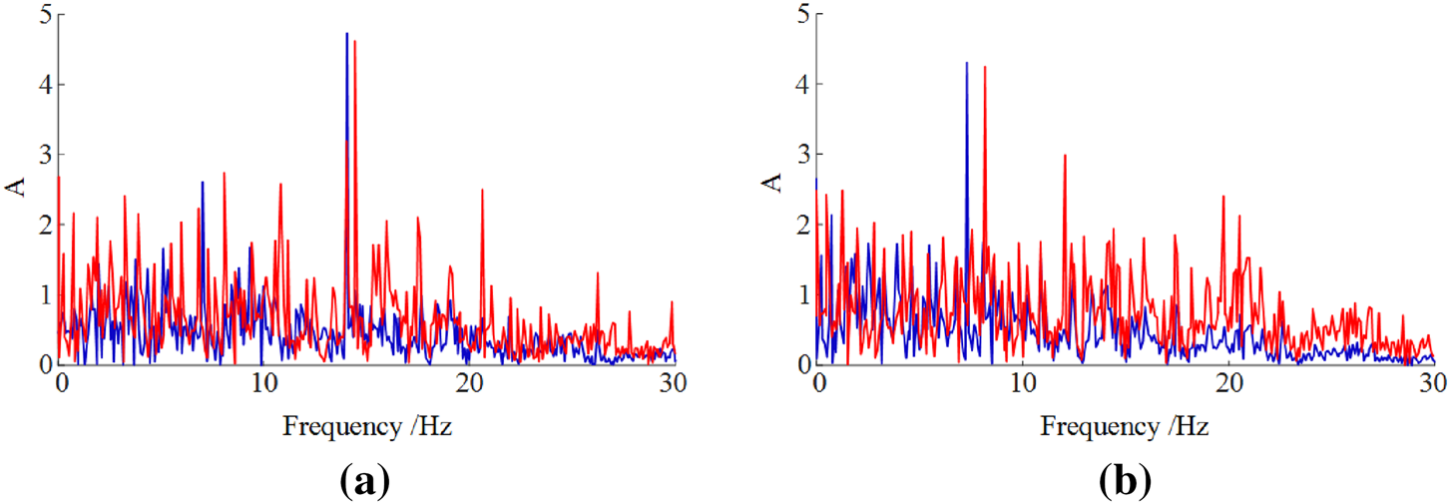
Vibration frequency spectra of rocker arm (**a**) and walking box (**b**).

As seen in Fig. 13, the simulation values of the main vibration frequency for rocker arm and walking box differ slightly from the experimental ones. The vibration spectra obtained from experiment have more other vibration frequencies than the simulated ones. As shown in Table 7, the simulated and experimental values of the main vibration frequency of rocker arm are 13.72 Hz and 14.24 Hz, respectively. The simulated and experimental values of the main vibration frequency of walking box are 7.98 Hz and 8.76 Hz, respectively. The error value for walking box is slightly less than 10%.

Then the simulated and experimental values of the vibration displacement for parts of the shearer under different working conditions are compared and analyzed. Table 8 shows the simulation value and experimental value of the vibration displacement of the key components under different traction speeds. Table 9 presents the simulated and experimental values of the vibration displacement of key components when f equals to 4. All but two relative errors of the vibration displacement between the simulated and experimental values for key parts of the shearer under different working conditions are less than 10%. For the two exceptions, the vibration displacement values of simulated and experimental ones are both less than 0.05 mm.

**Table 8:** Simulated and experimental vibration displacement values for parts of the shearer under different traction speeds.

*v* (m/min)	1.5	Relative error (%)	2	Relative error (%)	2.5	Relative error (%)	3	Relative error (%)	3.5	Relative error (%)
**Front rocker arm (mm)**										
Simulated	2.07	2.82	2.73	3.87	3.17	2.16	4.26	2.52	5.70	2.23
Experimental	2.13		2.84		3.24		4.37		5.83	
**Front traction part (mm)**										
Simulated	0.11	15.40	0.26	7.14	0.40	13.04	1.67	6.18	2.01	4.29
Experimental	0.13		0.28		0.46		1.78		2.10	
**Front walking box (mm)**										
Simulated	0.30	9.10	0.41	8.89	1.35	4.26	2.29	1.72	2.37	2.07
Experimental	0.33		0.45		1.41		2.33		2.42	
**Front support part (mm)**										
Simulated	0.30	3.26	0.30	9.10	0.32	3.03	0.33	2.94	0.35	5.41
Experimental	0.31		0.33		0.33		0.34		0.37	
**Machine body (mm)**										
Simulated	0.07	12.5	0.10	10.00	− 0.18	5.88	0.03	0	0.03	0
Experimental	0.08		0.11		− 0.17		0.03		0.03

**Table 9:** Simulated and experimental vibration displacement values for parts when f = 4.

*f* = 4	Simulated	Experimental	Relative error (%)
Front traction part (mm)	3.22	3.51	8.26
Front walking box (mm)	2.36	2.47	3.17
Front support part (mm)	1.87	2.02	7.43
Machine body (mm)	0.98	1.05	6.67

The reasons of errors are analyzed as follows: (1) in the theoretical model, the height error of the adjacent middle groove is not considered; (2) in the theoretical model, the influence of the vibration of the gear transmission system on the vibration of the whole machine is not considered; (3) in the theoretical model, the influence of coal gangue under the shearer shoes on the vibration of the whole machine is not considered; (4) in the theoretical model, the design sizes of the shearer rather than the actual ones are used for analysis, and thus the assembly errors of the machine produced in practical assembly process are not considered; (5) in the theoretical model, the influence of the vibration of electrical system and hydraulic system of the shearer on the vibration of the whole machine is not considered. (6) There are errors in the process of data processing.

## Conclusion

In order to study the vertical and pitch coupling dynamics characteristics of shearer under actual working conditions, the connection characteristics between key parts in existence of clearance and the support characteristics of the shearer have been comprehensively considered. A nonlinear vertical–pitch coupled dynamics model of the shearer with 13 degrees of freedom has been established. Experiments have been complemented to correct the drum loads, which has been applied as the external excitation of the model. Numerical methods have been used to analyze the dynamic characteristics of key parts of the shearer. The research results are as follows.

1. The shearer traction speed has been proposed to correct the drum loads. The correction coefficient has been determined and verified via experimental methods. The errors between the experimental results and the corrected drum loads in traction, vertical and axis directions were 0.77%, 2.67%, and 1.59%, respectively.
2. The vibration response curves of key parts of the shearer under the working conditions of a traction speed as 3 m/min, a mining pitch angle as 0° and a coal rock hardness coefficient (*f*) as 3 have been obtained. The vibration response curves of the front drum and rocker arm had larger fluctuation range than the others. The fluctuation ranges of vibration displacement and swing angle were − 0.4 to 0.8 rad and − 1 to 5 mm, respectively. The fluctuation range of the vibration acceleration for the front rocker arm was ± 300 mm/s^2^; the fluctuation range and fluctuation trend of the vibration response curves for traction part, walking box and supporting part were basically the same. The corresponding fluctuation ranges of vibration displacement were − 1 to 4.5 mm, 0–4 mm, and 0–4 mm, respectively. The fluctuation range of the vibration acceleration for front walking box was − 150 to 300 mm/s^2^. The vibration response frequencies of all key parts were low and their phase difference was small. When the vibration frequency was 13.72 Hz, the front rocker arm experienced the largest vibration response; when the vibration frequency was 7.98 Hz, the front walking box experienced the largest vibration response.
3. When the traction speed was 0–3 m/min, the change trends of vibration displacement for front rocker arm and front drum of the shearer were more obvious, and the change ranges of vibration swing angle were from 0.17 to 0.34 rad and from 0.15 to 0.32 rad for front drum and front walking box, respectively. The change range of vibration displacement for front walking box and traction part were from 0.30 to 2.37 mm and 0.11–2.01 mm, respectively. As coal rock solidity coefficient *f* increased from 3 to 5, the vibration displacement of all parts increased. The vibration swing angles for front drum and rocker arm varied in the range of 0.32–0.48 rad and 0.29–0.42 rad, respectively; the change ranges of vibration displacement for front traction part, walking box and support part were 0.50–4.20 mm, 1.96–4.64 mm and 1.00–4.00 mm, respectively. When the mining pitch angle increased from 0° to 30°, the vibration displacement and swing angle of front drum, front rocker arm and front walking box increased at a higher rate, and the change ranges of vibration displacement for walking box and swing angle for front roller and rocker arm were 2.29–4.32 mm, 0.36–0.52 rad and 0.27–0.50 rad, respectively.
4. Experimental method has been used to verify the solution results. Simulated and experiment values of the vibration response for key parts of the shearer under different working conditions have been compared and analyzed. Under the influence of the difference between the simulation solution assumptions and the experimental circumstances, the error between simulated and the experimental values were less than 10% for most of the cases. Only a few vibration displacement values had errors more than 10%, but the error values were less than 0.05 mm. Those results verified the accuracy of the established vertical–pitch coupled dynamics model of the shearer.

The investigation results can provide the guidance for the optimization control on operational parameters of the shearer, the theoretical basis for reliable operation and key parts fatigue life prediction and the theoretical guidance for manual operation of the shearer.

## Supplementary Information

Supplementary Information.
