# Supplementary material for: Analysis on vertical–pitch coupled dynamics characteristics of shearer with corrected load
Source: Sci Rep. 2021 Sep 20;11:18659. doi: 10.1038/s41598-021-98221-3 (PMC8452628; doi:10.1038/s41598-021-98221-3)
Supplement: Supplementary file 1 — Supplementary Information. [file 41598_2021_98221_MOESM1_ESM.doc]

**Appendix**

Matrix ***M*** was:

$$\mathbf{M}=\left[\begin{matrix} \mathbf{M}_{\mathbf{1}} & \mathbf{M}_{\mathbf{3}} \\ \mathbf{M}_{\mathbf{4}} & \mathbf{M}_{\mathbf{2}} \end{matrix}\right]$$

among them,

$\mathbf{M}_{\mathbf{1}}=\left[\begin{matrix} m_{1}+m_{2} & & & & & & \\ & m_{3} & & & & & \\ & & m_{5} & & & & \\ & & & m_{6} & & & \\ & & & & m_{7} & & \\ & & & & & m_{8} & \\ & & & & & & m_{10}+m_{11} \end{matrix}\right]$；$\mathbf{M}_{\mathbf{2}}=\left[\begin{matrix} m_{12} & & & & & \\ & m_{13} & & & & \\ & & m_{1}\cdot p^{2} & & & \\ & & & m_{11}\cdot p^{2} & & \\ & & & & I_{y3} & \\ & & & & & I_{y7} \end{matrix}\right]$；

$\mathbf{M}_{\mathbf{3}}=\left[\begin{matrix} 0 & 0 & -m_{1}\cdot p\cdot\sin\alpha_{1} & 0 & 0 & 0 \\ 0 & 0 & 0 & 0 & 0 & 0 \\ 0 & 0 & 0 & 0 & 0 & 0 \\ 0 & 0 & 0 & 0 & 0 & 0 \\ 0 & 0 & 0 & 0 & 0 & 0 \\ 0 & 0 & 0 & 0 & 0 & 0 \\ 0 & 0 & 0 & -m_{11}\cdot p\cdot\sin\alpha_{11} & 0 & 0 \end{matrix}\right]$；$\mathbf{M}_{\mathbf{4}}=\left[\begin{matrix} 0 & 0 & 0 & 0 & 0 & 0 & 0 \\ 0 & 0 & 0 & 0 & 0 & 0 & 0 \\ -m_{1}\cdot p\cdot\sin\alpha_{1} & 0 & 0 & 0 & 0 & 0 & 0 \\ 0 & 0 & 0 & 0 & 0 & 0 & -m_{11}\cdot p\cdot\sin\alpha_{11} \\ 0 & 0 & 0 & 0 & 0 & 0 & 0 \\ 0 & 0 & 0 & 0 & 0 & 0 & 0 \end{matrix}\right]$；

Matrix ***C*** was:

$$\mathbf{C}=\left[\begin{matrix} \mathbf{C}_{\mathbf{1}} & \mathbf{C}_{\mathbf{3}} \\ \mathbf{C}_{\mathbf{4}} & \mathbf{C}_{\mathbf{2}} \end{matrix}\right]$$

among them,

$\mathbf{C}_{\mathbf{1}}=\left[\begin{matrix} c_{qby} & -c_{qby} & 0 & 0 & 0 & 0 \\ -c_{qby} & C_{2,2} & -c_{zy5} & C_{2,4} & C_{2,5} & 0 \\ 0 & -c_{zy5} & c_{qpy}+c_{zy5} & 0 & 0 & 0 \\ 0 & C_{4,2} & 0 & C_{4,4} & C_{4,5} & 0 \\ 0 & C_{5,2} & 0 & C_{5,4} & C_{5,5} & -c_{zy8} \\ 0 & 0 & 0 & 0 & -c_{zy8} & c_{hpy}+c_{zy8} \end{matrix}\right]$；$\mathbf{C}_{\mathbf{2}}=\left[\begin{matrix} c_{hby} & 0 & 0 & 0 & 0 & 0 & -\frac{1}{2}c_{hpy}\cdot r \\ 0 & c_{qpy}+c_{zy12} & 0 & 0 & 0 & 0 & 0 \\ 0 & 0 & c_{hdy}+c_{zy13} & 0 & 0 & 0 & 0 \\ 0 & 0 & 0 & 0 & 0 & 0 & 0 \\ 0 & 0 & 0 & 0 & 0 & 0 & 0 \\ 0 & 0 & 0 & 0 & 0 & C_{12,12} & C_{12,13} \\ -\frac{1}{2}c_{hpy}\cdot r & 0 & 0 & 0 & 0 & C_{13,12} & C_{13,13} \end{matrix}\right]$；

$\mathbf{C}_{\mathbf{3}}=\left[\begin{matrix} 0 & 0 & 0 & 0 & 0 & -\frac{1}{2}r\cdot c_{qby} & 0 \\ 0 & -c_{zy12} & 0 & 0 & 0 & C_{2,12} & C_{2,13} \\ 0 & 0 & 0 & 0 & 0 & 0 & 0 \\ 0 & 0 & 0 & 0 & 0 & C_{4,12} & C_{4,13} \\ -c_{hby} & 0 & -c_{zy13} & 0 & 0 & C_{5,12} & C_{5,13} \\ 0 & 0 & 0 & 0 & 0 & 0 & 0 \end{matrix}\right]$；$\mathbf{C}_{4}=\left[\begin{matrix} 0 & 0 & 0 & 0 & -c_{hby} & 0 \\ 0 & -c_{zy12} & 0 & 0 & 0 & 0 \\ 0 & 0 & 0 & 0 & -c_{zy13} & 0 \\ 0 & 0 & 0 & 0 & 0 & 0 \\ 0 & 0 & 0 & 0 & 0 & 0 \\ -\frac{1}{2}r\cdot c_{qby} & C_{12,2} & 0 & C_{12,4} & C_{12,5} & 0 \\ 0 & C_{13,2} & 0 & C_{13,4} & C_{13,5} & 0 \end{matrix}\right]$；

$C_{2,2}=c_{cks}+c_{ckx}+c_{cms}+c_{cmx}+c_{qby}+c_{zy5}+c_{zy12}$；$C_{2,4}=-\left(c_{cks}+c_{ckx}+c_{cms}+c_{cmx}\right)$；$C_{2,5}=c_{cks}+c_{ckx}+c_{cms}+c_{cmx}$；

$C_{2,12}=\left(c_{cks}\cdot l_{cks}+c_{ckx}\cdot l_{ckx}+c_{cms}\cdot l_{cms}+c_{cmx}\cdot l_{cmx}+c_{qby}\cdot r\right)/2$；$C_{2,13}=\left(c_{cks}\cdot l_{cks}+c_{ckx}\cdot l_{ckx}+c_{cms}\cdot l_{cms}+c_{cmx}\cdot l_{cmx}\right)/2$；

$C_{4,2}=-\left(c_{cks}+c_{ckx}+c_{cms}+c_{cmx}\right)$；$C_{4,4}=c_{cks}+c_{ckx}+c_{cms}+c_{cmx}$；$C_{4,5}=-\left(c_{cks}+c_{ckx}+c_{cms}+c_{cmx}\right)$；

$C_{4,12}=-\left(c_{cks}\cdot l_{cks}+c_{ckx}\cdot l_{ckx}+c_{cms}\cdot l_{cms}+c_{cmx}\cdot l_{cmx}\right)/2$；$C_{4,13}=-\left(c_{cks}\cdot l_{cks}+c_{ckx}\cdot l_{ckx}+c_{cms}\cdot l_{cms}+c_{cmx}\cdot l_{cmx}\right)/2$；

$C_{5,2}=c_{cks}+c_{ckx}+c_{cms}+c_{cmx}$；$C_{5,4}=-\left(c_{cks}+c_{ckx}+c_{cms}+c_{cmx}\right)$；$C_{5,5}=c_{cks}+c_{ckx}+c_{cms}+c_{cmx}+c_{hby}+c_{zy8}+c_{zy13}$；

$C_{5,12}=\left(c_{cks}\cdot l_{cks}+c_{ckx}\cdot l_{ckx}+c_{cms}\cdot l_{cms}+c_{cmx}\cdot l_{cmx}\right)/2$；$C_{5,13}=\left(c_{cks}\cdot l_{cks}+c_{ckx}\cdot l_{ckx}+c_{cms}\cdot l_{cms}+c_{cmx}\cdot l_{cmx}+c_{hby}\cdot r\right)/2$；

$C_{12,2}=\left(c_{cks}\cdot l_{cks}+c_{ckx}\cdot l_{ckx}+c_{cms}\cdot l_{cms}+c_{cmx}\cdot l_{cmx}+c_{qby}\cdot r\right)/2$；$C_{12,4}=-\left(c_{cks}\cdot l_{cks}+c_{ckx}\cdot l_{ckx}+c_{cms}\cdot l_{cms}+c_{cmx}\cdot l_{cmx}\right)/2$；

$C_{12,5}=\left(c_{cks}\cdot l_{cks}+c_{ckx}\cdot l_{ckx}+c_{cms}\cdot l_{cms}+c_{cmx}\cdot l_{cmx}\right)/2$；$C_{12,12}=\left(c_{cks}\cdot l_{cks}^{2}+c_{ckx}\cdot l_{ckx}^{2}+c_{cms}\cdot l_{cms}^{2}+c_{cmx}\cdot l_{cmx}^{2}+c_{qby}\cdot r^{2}\right)/4$；

$C_{12,13}=\left(c_{cks}\cdot l_{cks}^{2}+c_{ckx}\cdot l_{ckx}^{2}+c_{cms}\cdot l_{cms}^{2}+c_{cmx}\cdot l_{cmx}^{2}\right)/4$；$C_{13,2}=\left(c_{cks}\cdot l_{cks}+c_{ckx}\cdot l_{ckx}+c_{cms}\cdot l_{cms}+c_{cmx}\cdot l_{cmx}\right)/2$；

$C_{13,4}=-\left(c_{cks}\cdot l_{cks}+c_{ckx}\cdot l_{ckx}+c_{cms}\cdot l_{cms}+c_{cmx}\cdot l_{cmx}\right)/2$；$C_{13,5}=\left(c_{cks}\cdot l_{cks}+c_{ckx}\cdot l_{ckx}+c_{cms}\cdot l_{cms}+c_{cmx}\cdot l_{cmx}+c_{hby}\cdot r\right)/2$；

$C_{13,12}=\left(c_{cks}\cdot l_{cks}^{2}+c_{ckx}\cdot l_{ckx}^{2}+c_{cms}\cdot l_{cms}^{2}+c_{cmx}\cdot l_{cmx}^{2}\right)/4$；$C_{13,13}=\frac{1}{4}\left(c_{cks}\cdot l_{cks}^{2}+c_{ckx}\cdot l_{ckx}^{2}+c_{cms}\cdot l_{cms}^{2}+c_{cmx}\cdot l_{cmx}^{2}+c_{hby}\cdot r^{2}\right)$。

Matrix ***K*** was:

$$\mathbf{K}=\left[\begin{matrix} \mathbf{K}_{\mathbf{1}} & \mathbf{K}_{\mathbf{3}} \\ \mathbf{K}_{\mathbf{4}} & \mathbf{K}_{\mathbf{2}} \end{matrix}\right]$$

among them,

$\mathbf{K}_{\mathbf{1}}=\left[\begin{matrix} k_{qby} & -k_{qby} & 0 & 0 & 0 & 0 \\ -k_{qby} & K_{2,2} & -k_{zy5} & K_{2,4} & K_{2,5} & 0 \\ 0 & -k_{zy5} & k_{qpy}+k_{zy5} & 0 & 0 & 0 \\ 0 & K_{4,2} & 0 & K_{4,4} & K_{4,5} & 0 \\ 0 & K_{5,2} & 0 & K_{5,4} & K_{5,5} & -k_{zy8} \\ 0 & 0 & 0 & 0 & -k_{zy8} & k_{hpy}+k_{zy8} \end{matrix}\right]$；$\mathbf{K}_{\mathbf{2}}=\left[\begin{matrix} k_{hby} & 0 & 0 & 0 & 0 & 0 & -\frac{1}{2}k_{hpy}\cdot r \\ 0 & k_{qpy}+k_{zy12} & 0 & 0 & 0 & 0 & 0 \\ 0 & 0 & k_{hdy}+k_{zy13} & 0 & 0 & 0 & 0 \\ 0 & 0 & 0 & 0 & 0 & 0 & 0 \\ 0 & 0 & 0 & 0 & 0 & 0 & 0 \\ 0 & 0 & 0 & 0 & 0 & K_{12,12} & K_{12,13} \\ -\frac{1}{2}k_{hpy}\cdot r & 0 & 0 & 0 & 0 & K_{13,12} & K_{13,13} \end{matrix}\right]$；

$\mathbf{K}_{\mathbf{3}}=\left[\begin{matrix} 0 & 0 & 0 & 0 & 0 & -\frac{1}{2}r\cdot k_{qby} & 0 \\ 0 & -k_{zy12} & 0 & 0 & 0 & K_{2,12} & K_{2,13} \\ 0 & 0 & 0 & 0 & 0 & 0 & 0 \\ 0 & 0 & 0 & 0 & 0 & K_{4,12} & K_{4,13} \\ -k_{hby} & 0 & -k_{zy13} & 0 & 0 & K_{5,12} & K_{5,13} \\ 0 & 0 & 0 & 0 & 0 & 0 & 0 \end{matrix}\right]$；$\mathbf{K}_{\mathbf{4}}=\left[\begin{matrix} 0 & 0 & 0 & 0 & -k_{hby} & 0 \\ 0 & -k_{zy12} & 0 & 0 & 0 & 0 \\ 0 & 0 & 0 & 0 & -k_{zy13} & 0 \\ 0 & 0 & 0 & 0 & 0 & 0 \\ 0 & 0 & 0 & 0 & 0 & 0 \\ -\frac{1}{2}r\cdot k_{qby} & K_{12,2} & 0 & K_{12,4} & K_{12,5} & 0 \\ 0 & K_{13,2} & 0 & K_{13,4} & K_{13,5} & 0 \end{matrix}\right]$；

$K_{2,2}=k_{cks}+k_{ckx}+k_{cms}+k_{cmx}+k_{qby}+k_{zy5}+k_{zy12}$；$K_{2,4}=-\left(k_{cks}+k_{ckx}+k_{cms}+k_{cmx}\right)$；$K_{2,5}=k_{cks}+k_{ckx}+k_{cms}+k_{cmx}$；

$K_{2,12}=\left(k_{cks}\cdot l_{cks}+k_{ckx}\cdot l_{ckx}+k_{cms}\cdot l_{cms}+k_{cmx}\cdot l_{cmx}+k_{qby}\cdot r\right)/2$；$K_{2,13}=\left(k_{cks}\cdot l_{cks}+k_{ckx}\cdot l_{ckx}+k_{cms}\cdot l_{cms}+k_{cmx}\cdot l_{cmx}\right)/2$；

$K_{4,2}=-\left(k_{cks}+k_{ckx}+k_{cms}+k_{cmx}\right)$；$K_{4,4}=k_{cks}+k_{ckx}+k_{cms}+k_{cmx}$；$K_{4,5}=-\left(k_{cks}+k_{ckx}+k_{cms}+k_{cmx}\right)$；

$K_{4,12}=-\left(k_{cks}\cdot l_{cks}+k_{ckx}\cdot l_{ckx}+k_{cms}\cdot l_{cms}+k_{cmx}\cdot l_{cmx}\right)/2$；$K_{4,13}=-\left(k_{cks}\cdot l_{cks}+k_{ckx}\cdot l_{ckx}+k_{cms}\cdot l_{cms}+k_{cmx}\cdot l_{cmx}\right)/2$；

$K_{5,2}=k_{cks}+k_{ckx}+k_{cms}+k_{cmx}$；$K_{5,4}=-\left(k_{cks}+k_{ckx}+k_{cms}+k_{cmx}\right)$；$K_{5,5}=k_{cks}+k_{ckx}+k_{cms}+k_{cmx}+k_{hby}+k_{zy8}+k_{zy13}$；

$K_{5,12}=\left(k_{cks}\cdot l_{cks}+k_{ckx}\cdot l_{ckx}+k_{cms}\cdot l_{cms}+k_{cmx}\cdot l_{cmx}\right)/2$；$K_{5,13}=\left(k_{cks}\cdot l_{cks}+k_{ckx}\cdot l_{ckx}+k_{cms}\cdot l_{cms}+k_{cmx}\cdot l_{cmx}+k_{hby}\cdot r\right)/2$；

$K_{12,2}=\left(k_{cks}\cdot l_{cks}+k_{ckx}\cdot l_{ckx}+k_{cms}\cdot l_{cms}+k_{cmx}\cdot l_{cmx}+k_{qby}\cdot r\right)/2$；$K_{12,4}=-\left(k_{cks}\cdot l_{cks}+k_{ckx}\cdot l_{ckx}+k_{cms}\cdot l_{cms}+k_{cmx}\cdot l_{cmx}\right)/2$；

$K_{12,5}=\left(k_{cks}\cdot l_{cks}+k_{ckx}\cdot l_{ckx}+k_{cms}\cdot l_{cms}+k_{cmx}\cdot l_{cmx}\right)/2$；$K_{12,12}=\left(k_{cks}\cdot l_{cks}^{2}+k_{ckx}\cdot l_{ckx}^{2}+k_{cms}\cdot l_{cms}^{2}+k_{cmx}\cdot l_{cmx}^{2}+k_{qby}\cdot r^{2}\right)/4$；

$K_{12,13}=\left(k_{cks}\cdot l_{cks}^{2}+k_{ckx}\cdot l_{ckx}^{2}+k_{cms}\cdot l_{cms}^{2}+k_{cmx}\cdot l_{cmx}^{2}\right)/4$；$K_{13,2}=\left(k_{cks}\cdot l_{cks}+k_{ckx}\cdot l_{ckx}+k_{cms}\cdot l_{cms}+k_{cmx}\cdot l_{cmx}\right)/2$；

$K_{13,4}=-\left(k_{cks}\cdot l_{cks}+k_{ckx}\cdot l_{ckx}+k_{cms}\cdot l_{cms}+k_{cmx}\cdot l_{cmx}\right)/2$；$K_{13,5}=\left(k_{cks}\cdot l_{cks}+k_{ckx}\cdot l_{ckx}+k_{cms}\cdot l_{cms}+k_{cmx}\cdot l_{cmx}+k_{hby}\cdot r\right)/2$；

$K_{13,12}=\left(k_{cks}\cdot l_{cks}^{2}+k_{ckx}\cdot l_{ckx}^{2}+k_{cms}\cdot l_{cms}^{2}+k_{cmx}\cdot l_{cmx}^{2}\right)/4$；$K_{13,13}=\left(k_{cks}\cdot l_{cks}^{2}+k_{ckx}\cdot l_{ckx}^{2}+k_{cms}\cdot l_{cms}^{2}+k_{cmx}\cdot l_{cmx}^{2}+k_{hby}\cdot r^{2}\right)/4$。

Matrix ***Y*** was:

$$\mathbf{Y}=\left[y_{2},y_{3},y_{5},y_{6},y_{7},y_{8},y_{10},y_{12},y_{13},\beta_{1},\beta_{11},\eta_{y3},\eta_{y7}\right]^{T}$$

Matrix ***F*** was:

$$\mathbf{F}=\left[\begin{matrix} -k_{qby}\cdot d_{y2}/2 \\ k_{qby}\cdot d_{y2}/2 \\ 0 \\ 0 \\ k_{hby}\cdot d_{y10}/2 \\ 0 \\ -k_{hby}\cdot d_{y10}/2 \\ 0 \\ 0 \\ -R_{y1}\cdot p\cdot\cos\left(\alpha_{1}+\lambda\right)-R_{x1}\cdot p\cdot\sin\left(\alpha_{1}+\lambda\right) \\ R_{y11}\cdot p\cdot\cos\left(\alpha_{11}+\lambda\right)+R_{x11}\cdot p\cdot\sin\left(\alpha_{11}+\lambda\right) \\ k_{qby}\cdot r\cdot d_{y2}/4 \\ k_{hby}\cdot r\cdot d_{y10}/4 \end{matrix}\right]$$
